# Imaging the neural underpinnings of freezing of gait in Parkinson’s disease

**DOI:** 10.1016/j.nicl.2022.103123

**Published:** 2022-07-25

**Authors:** Michella M. Bardakan, Gereon R. Fink, Laura Zapparoli, Gabriella Bottini, Eraldo Paulesu, Peter H. Weiss

**Affiliations:** a Cognitive Neuroscience, Institute of Neuroscience and Medicine (INM-3), Forschungszentrum Jülich, Jülich, Germany; b Department of Neurology, University Hospital Cologne and Faculty of Medicine, University of Cologne, Cologne, Germany; c Department of Psychology and NeuroMi, University of Milano-Bicocca, Milan, Italy; d IRCCS Istituto Ortopedico Galeazzi, Milan, Italy; e Department of Brain and Behavioral Sciences, University of Pavia, Pavia, Italy

**Keywords:** Gait impairment, Virtual reality, Motor imagery, Locomotor regions, Neuroimaging, PD, Parkinson's disease, FoG, freezing of gait, MI, motor imagery, VR, virtual reality, FC, functional connectivity, BG, basal ganglia, GP, globus pallidus, PPN, pedunculopontine nucleus, MLR, mesencephalic locomotor region, SMA, supplementary motor area

## Abstract

Freezing of gait (FoG) is a paroxysmal and sporadic gait impairment that severely affects PD patients’ quality of life. This review summarizes current neuroimaging investigations that characterize the neural underpinnings of FoG in PD. The review presents and discusses the latest advances across multiple methodological domains that shed light on structural correlates, connectivity changes, and activation patterns associated with the different pathophysiological models of FoG in PD. Resting-state fMRI studies mainly report cortico-striatal decoupling and disruptions in connectivity along the dorsal stream of visuomotor processing, thus supporting the ‘interference’ and the ‘perceptual dysfunction’ models of FoG. Task-based MRI studies employing virtual reality and motor imagery paradigms reveal a disruption in functional connectivity between cortical and subcortical regions and an increased recruitment of parieto-occipital regions, thus corroborating the ‘interference’ and ‘perceptual dysfunction’ models of FoG. The main findings of fNIRS studies of actual gait primarily reveal increased recruitment of frontal areas during gait, supporting the ‘executive dysfunction’ model of FoG. Finally, we discuss how identifying the neural substrates of FoG may open new avenues to develop efficient treatment strategies.

## Highlights

• Review of recent (after 2012) imaging studies on Parkinsonian freezing of gait.
• Virtual reality studies report functional decoupling of cortico-striatal circuits.
• Motor imagery studies reveal increased recruitment of parieto-occipital regions.
• fNIRS studies converge on reporting higher activity within prefrontal regions.
• Imaging findings support pathophysiological models of freezing of gait.

## 1 Introduction

Freezing of gait (FoG) is a debilitating motor symptom that affects around 25% of patients with early PD (Perez-Lloret et al., 2014) and more than half of the patients with advanced PD (Giladi, 2001). It is considered a critical factor for falls in this population. FoG is defined as a brief, involuntary, and unexpected disruption of gait that persists for several seconds or longer, during which PD patients commonly report the feeling that their feet are ‘glued to the floor’ (Amboni et al., 2015). This paroxysmal gait disturbance is intimately linked to postural instability (Giladi et al., 2001) and executive dysfunction (Yogev-Seligmann et al., 2008) and is scarcely associated with the motor triad (tremor, bradykinesia, and rigidity). FoG can occur during gait initiation and regular walking. At gait initiation, FoG is often preceded by multiple deficits in anticipatory postural adjustments. In contrast, during regular walking, FoG is preceded by reduced step amplitude and increased step frequency (Grabli et al., 2013). Other common features of FoG include higher step-to-step variability (Hausdorff et al., 2003), step time variability (Weiss et al., 2015a), and step amplitude variability (Barbe et al., 2014).

Despite the clinical characteristics described above, FoG remains challenging to predict, mainly due to its sporadic nature and multiple freezing triggers (Nieuwboer and Giladi, 2013). There has been an attempt to divide common FoG triggers into three subgroups: motor, cognitive, and limbic (Ehgoetz Martens et al., 2018). Motor triggers of FoG include turning or navigating through narrow or confined spaces. Cognitive triggers include dual tasks and other forms of additional cognitive load during walking. Finally, limbic or affective triggers comprise internal factors about the mental state of PD patients, e.g., anxiety (Giladi and Hausdorff, 2006) or panic attacks (Lieberman, 2006).

Although FoG in PD patients is more prevalent when the patients are OFF dopaminergic treatment (60%) than when ON (36–38%) (Amboni et al., 2015), FoG shows, similarly to other gait disturbances that emerge in advanced PD stages, an intersubjective variability in responsiveness and sometimes even resistance to dopaminergic treatment (Nutt et al., 2011). Besides, FoG hardly responds to other treatment strategies, such as deep brain stimulation (DBS) (Castrioto et al., 2011). In contrast, a non-pharmacological treatment through visual or auditory cueing alleviates FoG symptoms (Nieuwboer et al., 2007). The positive effects of visual cues (e.g., dynamic vectorial visual patterns) on FoG point to a higher dependence on visual processing and increased attentional processing in PD patients to compensate for locomotion deficits (Azulay et al., 2006).

Notably, the prevalence of PD in our aging population is predicted to substantially increase by approximately 30% by 2030 (Chen et al., 2001), with a parallel increase in the socioeconomic burden. FoG shows an increasing prevalence across the different PD stages, with increasing detrimental effects on various aspects of the patients’ daily lives, significantly reducing the PD patients’ quality of life (QoL). Moreover, as mentioned before, FoG in advanced PD is often resistant to pharmacological treatment or deep brain stimulation (DBS). Thus, developing new strategies and interventions that efficiently target gait impairments, particularly FoG, is warranted in PD patients. A prerequisite for these endeavors is a better understanding of gait’s neural control in healthy people and its dysfunction in PD.

### 1.1 Functional neuroanatomy of human gait control

The knowledge about the neural mechanisms controlling human gait is still incomplete, mainly due to the complexity of the human locomotor system and the technical limitations of studying locomotion. Accordingly, the current knowledge about the neural control of human gait is primarily derived from animal models. Studies on gait regulation in animals revealed widespread neural circuitries spanning different nervous system levels, including cortical, subcortical, brainstem, and spinal cord structures (Takakusaki et al., 2008). However, comparisons between gait control mechanisms in animals and humans warrant caution since most animal studies investigate quadrupedal locomotion (e.g., in cats, monkeys, and rats), the neural mechanisms of which are likely to differ substantially from those relevant for bipedal locomotion (Nakazawa et al., 2012).

**Fig. 1:**
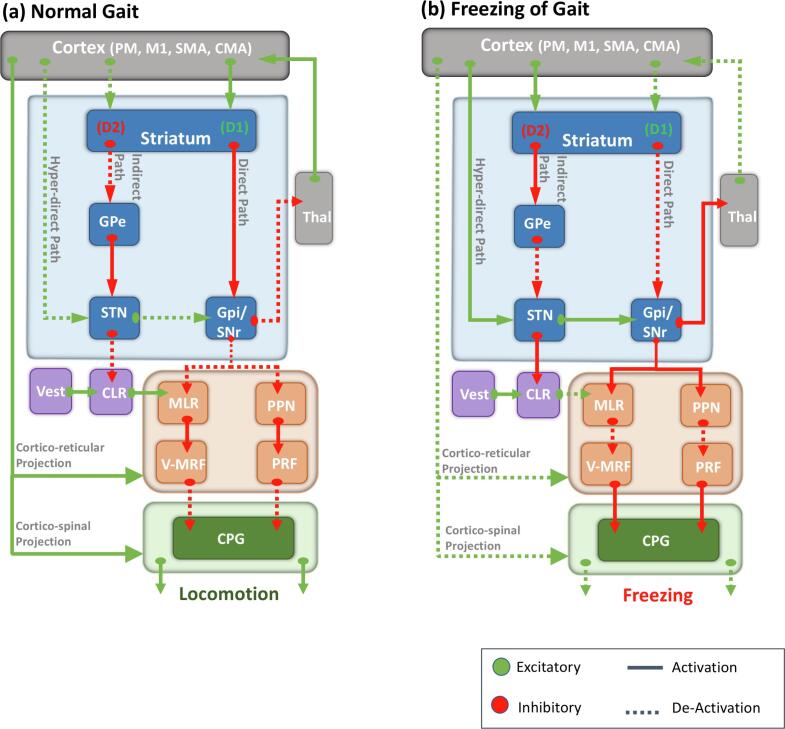
A physiological model of gait and pathophysiological model of freezing of gait (FoG). The normal control of gait requires coordinated activity between the cortex, the basal ganglia (BG), and the cerebellum to modulate the brainstem locomotor regions’ output. In a healthy system (a), an efficient initiation, execution, and termination of the desired movement are achieved via the excitation of the target motor plan (direct pathway) and the inhibition of competing motor plans (hyper-direct and indirect pathways). Before activating a target motor system, the baseline inhibition is increased in this system via the hyper-direct pathway that projects directly from the SMA to the STN. The target system is later released from this inhibition through its excitation via the striatum’s direct pathway to the GPi, resulting in the initiation and execution of the planned movement. The cessation of the movement is mediated by the target motor system’s re-inhibition via the indirect pathway from the striatum to the GPe and STN. This coordinated excitation and inhibition allow the BG to modulate the motor output of the locomotor regions in the brainstem and cerebellum to produce the desired movement. However, in Parkinson’s disease (b) this coordination is compromised, leading to a sustained over-inhibition (increased inhibition via the hyper-direct and indirect pathways, and decreased disinhibition via the direct pathway) of the brainstem structures via the GPi/SNr that hinders the initiation and execution of movement resulting in FoG. FoG can occur at the initiation stage (possibly due to sustained baseline inhibition via the hyper-direct pathway and decreased direct pathway disinhibition) or during an ongoing movement (possibly due to temporally abrupt re-inhibition via the indirect pathway and decreased disinhibition of direct pathway). *CLR: cerebellar locomotor region, CMA: cingulate motor area, CPG: central pattern generators, D1: excitatory dopamine receptor, D2: inhibitory dopamine receptor, GPe: globus pallidus externus, GPi: globus pallidus internus, M1: primary motor cortex, MLR: mesencephalic locomotor region, PM: premotor cortex, PPN: pedunculopontine nucleus, PRF: pontine reticular formation, SMA: supplementary motor area, SNr: substantia nigra pars reticulata, STN: subthalamic nucleus, Thal: thalamus, vMRF: ventromedial reticular formation.*

The neural networks that govern human gait are organized in an evolutionary hierarchy (see Fig. 1) that provides optimal gait control and high flexibility in adapting gait patterns to environmental conditions (Grabli et al., 2013). The peripheral nervous system, the musculoskeletal system, and the central locomotion pattern generators (CPGs) constitute the building blocks of gait control. These structures are primarily responsible for low-level automatic functions such as muscle tone regulation and the production of rhythmic, dynamic, and flexible locomotion (Hägglund et al., 2010). In the middle of the hierarchy reside the so-called ‘locomotor regions of the brain’ comprised of the cerebellar locomotor region (CLR), the subthalamic locomotor region (SLR), and the mesencephalic locomotor region (MLR), which is comprised of the pedunculopontine nucleus (PPN) and the cuneiform nucleus (CN). Their primary function is to activate and regulate the output of the CPGs associated with the initiation and cessation of movements and the regulation of the basic step cycle. Thereby, the locomotor regions are essential for producing and regulating effective lower-limb movements and maintaining posture and balance in response to the environment’s dynamic changes.

**Fig. 2:**
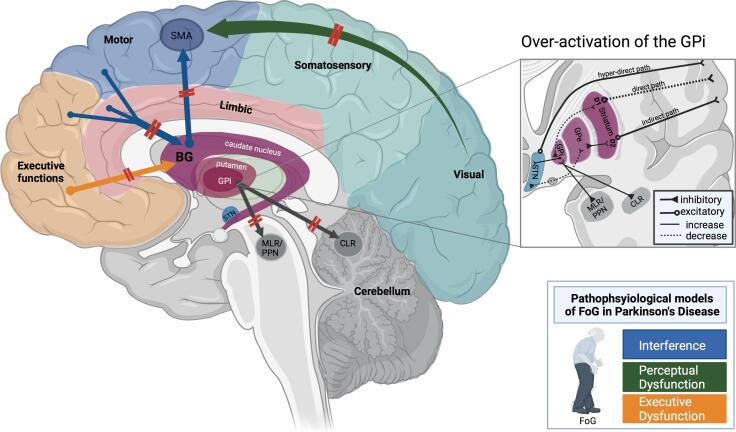
Schematic depiction of the main pathophysiological models of freezing of gait (FoG) in Parkinson’s disease. The interference model (presented by blue arrows) posits that FoG is due to a failure in crosstalk between several cortical areas (cognitive, motor, and limbic) and the basal ganglia (BG). This dysfunction in crosstalk exerts a higher load on the BG-SMA loop for internally-generated movements, which eventually leads to the disruption of the BG-SMA loop and the ensuing lack of gait automaticity. This model accounts for episodes of FoG that occur during multitasking while walking. The perceptual dysfunction model (presented by the green arrow) proposes that FoG results from a malfunction along the dorsal stream of visuomotor processing. Thus, visual input is not adequately transferred from occipital areas to somatosensory areas and later to frontal areas responsible for generating an appropriate motor plan. This model accounts for the failure in adapting the ongoing locomotion in response to changes in the environment (e.g., crossing doorways), which can manifest in an episode of FoG. The executive dysfunction model (presented by the orange arrow) considers FoG to result from a specific decoupling between cognitive areas in the frontal lobe and the BG. According to this model, executive control areas are heavily recruited to compensate for the lack of gait automaticity, which can account for FoG during cognitively demanding dual tasks, such as obstacle avoidance while walking. All these different mechanisms proposed by the models of FoG eventually overwhelm the limited processing capacity of the striatum in PD and consequently lead to a hyperactive GPi that overly inhibits brainstem (MLR/PPN) and cerebellar (CLR) structures, eventually producing episodes of FoG. Note that this schematic illustration does not cover the ‘decoupling’ and ‘abnormal gait pattern generation’ models of FoG, given that none of the discussed imaging studies provide corroborating results to support these models. (Figure created with BioRender.com) *BG: basal ganglia, CLR: cerebellar locomotor region, D1/2: dopamine receptors, FoG: freezing of gait, GPi/e: globus pallidus internus/externus, MLR: mesencephalic locomotor region, PPN: pedunculopontine nucleus, SMA: supplementary motor area, STN: subthalamic nucleus.*

The top of the gait control hierarchy comprises higher-level control centers in the cerebral cortex and the basal ganglia (BG) that modulate indirectly and directly the locomotor regions’ activity in generating efficient volitional gait regulation. At rest, the globus pallidus internus (GPi) and the substantia nigra reticularis (SNr) provide constant GABAergic inhibitory signals to the MLR/PPN that restrict the information flow in the spinal cord and prevent unwanted movements. During motor activity, input from frontoparietal areas can amplify or reduce these inhibitory signals (Lewis and Shine, 2016a). In particular, the execution of a motor plan requires a sequential regulation of the BG’s inhibitory output that permits an efficient initiation, execution, and termination of the selected motor plan via the hyper-direct, direct, and indirect pathways, respectively, that connect the cortex with the BG (see Fig. 1, Fig. 2). Several major cerebral areas indirectly contribute to the voluntary execution of a motor plan at the level of the brainstem through their connections with the BG (via the basalganglia-thalamo-cortical circuits) and the cerebellum (via the thalamus and pontine nuclei) (Hashimoto, 2006). For instance, the supplementary motor area (SMA) is involved in step initiation and mediating precisely organized alterations in posture and balance, called anticipatory postural adjustments (APA) (Jacobs et al., 2009a), preceding voluntary walking movements. In the later stages of processing the motor plan, the primary motor cortex (M1) contributes to the execution and coordination of precise lower-limb movements (Takakusaki, 2008). The posterior parietal cortex (PPC) generates a temporospatial map of the body’s position in the surrounding environment vital for efficient navigation in a novel or unfamiliar environment (Marigold and Drew, 2017, Nordin et al., 2019).

In conclusion, the neural systems underlying gait control in humans comprise complex mechanisms with intertwined structures, in which higher-order cortical areas indirectly regulate the output of the locomotor regions via established reciprocal connections with subcortical structures. This hierarchical bi-directional organization allows the human gait control system to adapt its motor output to changing intentions and environments flexibly. However, this flexible planning and execution of walking movements is deficient in PD patients since the underlying neurodegenerative disease process affects the gait control system at different levels.

### 1.2 Pathophysiological models of freezing of gait (FoG)

Five non-exclusive pathophysiological models have been proposed to explain the origin of FoG in PD (Nutt et al., 2011, Okuma et al., 2018). These models may account for FoG-related pathophysiological changes at different neural levels and in different contexts, thereby helping explain the apparent heterogeneity of FoG in PD patients (Nieuwboer and Giladi, 2013). Models of FoG depict the multilevel failures of the locomotor network associated with FoG, ranging from deficits in lower-level centers (i.e., the central pattern generators; CPG) to deficits in higher-level centers (i.e., the prefrontal cortex; PFC).

The (i) ‘*abnormal gait pattern generation*’ hypothesis proposes that the asymmetric and uncoordinated gait preceding episodes of freezing (Hausdorff et al., 2003) results from an abnormal control output from the CPG in the spinal cord. The (ii) ‘*disordered central drive and automaticity*’ hypothesis suggests that FoG is due to a disruption in the BG-SMA loop for internally initiated movements and the ensuing lack of gait automaticity. The reduced automaticity of gait is supposed to be compensated by pathological recruitment of higher-level cortical areas in PD patients suffering from FoG that, in turn, overwhelms the available cognitive resources and thus underlies the frequent episodes of FoG that occur during dual-task walking. The ‘*interference’* model extends this hypothesis, suggesting that FoG is due to deficient crosstalk between diverse cortical areas and the BG (i.e., not only between the SMA and the BG). Due to the deficient cortico-subcortical crosstalk, the signals from the cognitive, motor, and limbic cortical areas overwhelm the limited processing capacity of the striatum in PD patients and lead to an overinhibition of brainstem structures, eventually manifesting in FoG (Lewis and Barker, 2009, Lewis and Shine, 2016). Another hypothesis states that a (iii) ‘*decoupling*’ between posture preparation (SMA) and step initiation (the motor cortex) causes multiple dysfunctional anticipatory postural adjustments that are manifested as knee trembling during FoG (Jacobs et al., 2009b). The breakdown between gait and posture puts a higher load on the hyper-direct pathway (SMA-GPi). This extensive recruitment of the SMA-GPi-pathway is maladaptive as it leads to increased excitation of the inhibitory output nucleus GPi that eventually inhibits the locomotor regions and thus causes FoG. The (iv) ‘*perceptual dysfunction*’ hypothesis considers FoG to be the outcome of dysfunction along the dorsal visuomotor processing stream leading to deficits in online locomotor adaptation to environmental changes (Almeida and Lebold, 2010). This model accounts well for the observed reduction of gait speed during doorway crossing in PD patients with FoG (Cowie et al., 2012). Finally, the (v) ‘*executive dysfunction*’ view claims a specific decoupling between the frontal lobe and the BG to be the source of FoG (Amboni et al., 2008). According to this model, PD patients with FoG recruit executive function areas to overcome the loss of gait automaticity, which can account for episodes of FoG during multitasking in gait, such as obstacle avoidance.

The apparent heterogeneity of the mechanisms underlying FoG in PD proposed by the five non-exclusive pathophysiological models of FoG indicates that consensus on the neurological underpinnings of FoG is far from reach and, consequently, extensive research is warranted to unravel the complexities of this phenomenon. Studies employing imaging techniques contribute valuable new data for a better understanding of the physiology and pathophysiology of human gait control. Identifying major neural substrates of FoG may open new avenues for developing novel and efficient treatment strategies for FoG in PD patients. This review summarizes and critically evaluates the recent imaging studies on FoG in PD across multiple methodological domains to shed light on structural correlates, connectivity changes, and activation patterns associated with the pathophysiology of FoG in PD patients. Furthermore, the disparate imaging results are related to current pathophysiological models of FoG in PD. For a complementary review that discusses electrophysiological findings in light of the pathophysiological models of FoG, the reader is referred to the recent review by Marquez et al. (2020).

## 2 Method

The review encompasses articles not previously covered or thoroughly discussed by recent reviews on FoG in PD patients (Mirelman et al., 2019, Weiss et al., 2020). The incorporated articles’ eligibility criteria included peer-reviewed articles published in English between 2012 and 2021. Thus, the current review focuses on the papers published after the review by Maillet et al., 2012a, Maillet et al., 2012b that extensively covered all relevant imaging studies published before 2012 on FoG in PD. A systematic review of imaging studies of FoG in PD after 10 years is a reasonable endeavor given the advances in neuroimaging.

The PubMed database and Google Scholar were used to collect the articles with the search queries ‘Parkinson’ AND ‘Freezing of gait’ AND (‘functional MRI’ OR ‘structural MRI’ OR ‘fNIRS’ OR ‘resting-state MRI’ OR ‘task-based MRI’ OR ‘virtual reality’ OR ‘motor imagery’). After a systematic assessment of the articles’ eligibility, the total number of reported articles in this review is 40 articles. These articles were divided into 18 resting-state MRI studies (2 studies assessing structural connectivity, 12 studies assessing functional connectivity, and 4 studies assessing both structural and functional connectivity), 13 task-based MRI studies (6 studies employing virtual reality paradigms, 6 studies employing motor imagery paradigm, 1 study using a novel foot-flexion task), and 9 functional near-infrared spectroscopy (fNIRS) studies.

In order to further assess the quality of evidence provided by the different imaging studies, we systematically graded each reported study based on six pre-defined criteria, i.e., studied sample, clinical groups, FoG/gait assessment, correction for multiple comparisons, control for differences in covariates, and experimental design (one to two points for each criterion; please see the Tables S1, S2 and S3 in the supplementary materials). Studies gained or lost points based on the number of criteria they met or failed to meet. Thus, each study was assigned a grade that corresponded to the total number of points gained minus the total number of points deducted.

## 3 Structural and resting-state functional connectivity studies of FoG in Parkinson’s disease

### 3.1 Imaging studies of structural connectivity associated with FoG in Parkinson’s disease

MRI-studies of white matter (WM) structural connectivity investigate abnormalities in WM tracts by diffusion tensor imaging (DTI) and examine mainly mean diffusivity (Md) and fractional anisotropy (FA). Several findings have emerged from different WM connectivity studies, despite differences in experimental methods and analysis approaches, namely whole-brain voxel-wise analysis and region of interest (ROI) correlation analysis.

Several structural connectivity studies report patterns of WM abnormalities that constitute potential biomarkers supporting the ‘interference model’, i.e., deficient crosstalk between cortical areas and the BG. Notably, structural imaging studies revealed a distributed pattern of reduced WM tracts connecting subcortical structures, encompassing the cerebellum (Bharti et al., 2019a), subthalamic nucleus (Fling et al., 2014), and the pedunculopontine nucleus (PPN; Fling et al., 2013, Wang et al., 2016), with higher-level cortical areas in PD patients with FoG. Besides, structural connectivity studies reported a disruption in WM connectivity between motor, limbic, and cognitive structures in PD patients with FoG, most dominantly for frontoparietal connections (Canu et al., 2015, Pietracupa et al., 2018). Moreover, a trend emerged toward right hemispheric lateralization of the structural abnormalities associated with FoG (Canu et al., 2015, Fling et al., 2014, Fling et al., 2013, Pietracupa et al., 2018). Importantly, none of these studies related the observed right hemispheric lateralization of WM abnormalities to the clinical lateralization of PD-related motor deficits. For instance, Fling et al. (2013) reported reduced structural connectivity between the PPN and the thalamus, the cerebellum and the frontal cortex only in the right hemisphere of PD patients with FoG. However, these WM abnormalities were not correlated with the reported motor deficits that were more pronounced on the contralateral left part of the body in the studied sample of PD patients with FoG.

Other structural imaging studies align with the ‘executive dysfunction’ model of FoG, which proposes a decoupling between the frontal lobe and the BG. These studies report WM tract disruptions between frontal areas and subcortical structures (Fling et al., 2013, Wang et al., 2016) and distributed WM damage related to cognitive areas of the frontal lobe (Canu et al., 2015). Note that these findings may have been biased by concurrent cognitive impairments in the studied sample of PD patients suffering from FoG, since the examined PD patients with FoG exhibited severe cognitive impairments across different neuropsychological domains compared to healthy participants and PD patients without FoG. For instance, the sample of PD patients with FoG investigated in Canu et al.'s (2015) study incorporated a substantial number of patients with mild cognitive impairment (MCI). Notably, their PD patients with FoG, who scored worse on tests assessing visuospatial functions, also exhibited WM damage in the right parietal cortex compared to PD patients without FoG. This suggests that the observed structural abnormalities may not be specific to FoG *per se* but may be related to other confounding factors such as cognitive impairment and general (motor) deficits associated with Parkinson’s disease.

**Table 1:** Structural and resting-state functional connectivity studies investigating freezing of gait (FoG) in Parkinson’s disease (PD) patients.

Author/Year	Sample	Dopaminergic status	Clinical/neuropsychological parameters and FoG/gait assessment	Imaging modality and parameters	Experimental design	Main findings in PD patients with FoG	Supported Model(s)
Bharti et al. (2019a)	15 FoG+ 16 FoG- 16 HC	OFF	H&Y, MMSE, UPDRS-III, HAM-D, FoG-Q and TUG	Structural DTI (ROI) and Functional connectivity	Cross-sectional Correlational (indirect with FoG-Q scores)	Lower WM connectivity of the CRB with supratentorial brain structures (C/T FoG- & HC) correlates positively with FoG-Q. Lower FC of the dentate nucleus with the brainstem, right BG, & fronto-parieto-occipital cortices (C/T FoG-), and higher FC of the CLR with cerebellar areas (C/T HC) correlate positively with FoG-Q	Interference AND/OR Perceptual Dysfunction
Bharti *et al.,* (2019b)	15 FoG+ 16 FoG- 16 HC	OFF	H&Y, MMSE, LEDD, UPDRS-III, HAM-D, FoG-Q and TUG	Functional connectivity (FC) within and between networks (ICA)	Cross-sectional Correlational (indirect with FoG-Q scores)	Higher FC within the right fronto-parietal, frontal & BG networks (C/T FoG-) and lower FC between right fronto-parietal and executive control RSNs (C/T HC) correlate negatively with FoG-Q	Interference
Canu et al. (2015)	22 FoG+ (13 MCI) 35 HC Independent sample of: 28 FoG+ (14 MCI) 25 FoG- (10 MCI) 30 HC	OFF ON (for the independent sample)	H&Y, MMSE, LEDD, UPDRS-III, ACE-R, BADA, BBS, BDI, MCI, RAVLT, TMT, FoG-Q, TUG, and 10 m walking test. FoG + in the independent sample: worse scores on visuospatial tests	Structural DTI (findings replicated in an independent sample) and Functional connectivity (FC)	Cross-sectional Correlational (indirect with UPDRS-III scores)	Distributed WM damage in the CRB, sensorimotor & cognitive areas C/T HC (in both samples) and in the right parietal cortex & CC C/T FoG- (in the independent sample). Lower FC within sensorimotor areas (M1 and SMA), frontoparietal regions, and occipital cortex (C/T HC)	Executive Dysfunction AND/OR Perceptual Dysfunction
Droby et al. (2021)	16 FoG+ 15 FoG- 20 HC	ON	H&Y, UPDRS-III, MoCA and FoG-Q. FoG+: worse performance on MoCA visuospatial and attention scores	Functional connectivity (FC) Whole-brain FC changes within midbrain inter-connected regions. Voxel-based morphometry	Cross-sectional Correlational (indirect with FoG-Q scores)	Lower FC in parieto-temporal cortex (C/T HC) and in the mid-cingulate (C/T FoG-). In FoG+: FC in right PCG correlated negatively with FoG-Q, and the FC in the left ACC, SMA, & right cerebellar crus positively correlated with MoCA.	Interference
Fling et al. (2013)	14 FoG+ 12 FoG- 15 HC	OFF	H&Y, UPDRS, Eriksen flanker task, MoCA, Stroop task, FoG-Q, TUG, and clockwise vs anticlockwise turning. FoG+: lower scores on MoCA and on the Stroop task	Structural DTI (ROI)	Cross-sectional Correlational (indirect with Stroop task)	Reduced right hemisphere PPT connectivity to CRB, thalamus, frontal & prefrontal areas (C/T HC & FoG-)	Executive Dysfunction
Fling et al. (2014)	8 FoG+ 7 FoG- 14 HC	OFF	H&Y, UPDRS, new FoG-Q, and a gait task: single and dual-task clockwise vs anticlockwise turning.	Structural DTI (ROI) and Functional connectivity (FC)	Cross-sectional Correlational (indirect with behavioral measures of gait and with FoG-Q)	Lower WM connectivity in the right STN-SMA loop (C/T HC) Higher FC between the SMA & left CLR, and between SMA & bilateral MLR (C/T FoG- & HC). The latter positively correlated with FoG-Q and behavioral measures of gait	Interference
Jung et al. (2020)	26 FoG+ (FoG-vulnerable) 61 FoG- (FoG-resistant) 27 HC	OFF	MMSE, UPDRS-III, BDI and CCSIT. Assessment for the presence of FoG every 3–6 months by gait experts.	Functional connectivity (FC) between the motor cerebellum and the whole brain	Longitudinal (5 years from MRI scan) Cross-sectional Correlational (indirect with FoG latency: time of symptoms onset)	Increased FC between the CRB and parieto-occipito-temporal association cortices [including the right SPL, left ITG, right MTG, and right MOG (C/T FoG- & HC)] correlate negatively with FoG latency	Perceptual Dysfunction
Lench et al. (2020)	27 FoG+ 27 FoG-	ON	LEDD, UPDRS I-IV, new FoG-Q and TUG (single and dual task)	Functional connectivity (FC) Whole brain seed-to-voxel analysis	Cross-sectional Correlational (indirect with TUG scores)	Higher FC of left MLR/PPN to sensori-motor regions [left temporal lobe, bilateral midcingulate cortex, & SMG (C/T FoG-)] correlate positively with longer turning duration	Interference
Lenka et al. (2015)	15 FoG+ 13 FoG- 30 HC	Not specified	H&Y, LEDD, MMSE, UPDRS and FoG-Q	Functional connectivity (FC) Whole brain seed-to-voxel analysis	Cross-sectional Correlational (indirect with FoG-Q scores)	Lower interhemispheric FC of left parietal cortex with auditory & primary somatosensory areas (C/T FoG-) correlate negatively with FoG-Q	Perceptual Dysfunction
Miranda-Domínguez et al. (2020)	13 FoG+ 14 FoG- 16 HC	OFF	LEDD, UPDRS-III, MoCA, FoG-Q, and a gait task: walking, clockwise and anticlockwise turning.	Functional connectivity (FC) Whole brain and ROI seed-based analyses	Cross-sectional Correlational (indirect with behavioral measures of gait)	Higher FC between left GP and primary somatosensory cortex (C/T FoG-) correlate positively with behavioral measures of FoG. Lower FC between the left vestibular cortex and regions of the DMN (C/T FoG-)	Interference AND/OR Perceptual Dysfunction
Pietracupa et al. (2018)	21 FoG+ 16 FoG- 19 HC	OFF	H&Y, LEDD, MMSE, UPDRS-III, FAB, HAM-D, FoG-Q and TUG	Structural DTI	Cross-sectional Correlational (indirect with FoG-Q and neuropsycho-logical test scores)	Widespread WM disruption [predominantly in the right hemisphere between motor, cognitive & limbic structures (C/T HC)] correlates positively with cognitive scores (FAB)	Interference
Potvin-Desrochers et al. (2019)	14 FoG+ 13 FoG-	ON	H&Y, UPDRS-III, HADS, MoCA, and new FOG-Q	Functional connectivity (FC) Subcortical structures seed- to-voxel analysis	Cross-sectional	Higher FC of bilateral thalamus and bilateral GPe with visual areas (C/T FoG-)	Perceptual Dysfunction
Steidel et al. (2021)	27 FoG+ 32 FoG-	OFF	H&Y, LEDD, MMSE, PANDA, PDQ39, UPDRS-III, BDI-II, tests for attention, memory, language, executive & visuospatial functions, and FoG-Q	Functional connectivity (FC), seed-based analysis (left caudate cluster or ventral tegmental area, VTA)	Cross-sectional Correlational (indirect with FoG-Q scores and dopaminergic degeneration)	Higher FC between bilateral VTA & DMN regions and higher FC between left caudate & right visual cortex (C/T FoG-) correlate negatively with dopamine levels. In all PD: positive correlation of FoG-Q and FC of VTA with DMN regions.	Interference AND/OR Perceptual Dysfunction
Tessitore et al. (2012)	16 FoG+ 13 FoG- 15 HC	ON	MMSE, UPDRS I-IV, BDI, CA, FAB, RCPM, TPCT, VFT, Corsi block span, verbal spa, Stroop task, and FoG-Q FoG+: lower scores on frontal lobe functions	Functional connectivity (FC) ICA	Cross-sectional Correlational (indirect with FoG-Q scores)	Lower FC within executive attention network (right middle frontal & angular gyrus) and visual network (right occipito-temporal gyrus; C/T FoG-) correlate negatively with FoG-Q	Perceptual Dysfunction AND/OR Executive Dysfunction
Vervoort et al. (2016)	13 FoG+ 60 FoG- 20 HC	OFF	H&Y, LEDD, MMSE, ANT, AIT, FAB, MoCa, TMT, new FoG-Q, and a gait task: single vs dual task turning	Functional connectivity (FC) ROI within fronto-parietal and motor network	Cross-sectional Correlational (indirect with behavioral measures of turning)	Lower FC within striatum and between caudate & STL as well as higher FC between dorsal putamen & precuneus (C/T FoG-) correlate with worse DT turning	Interference
Wang et al. (2016)	14 FoG+ 16 FoG- 16 HC	OFF	LEDD, MMSE, UPDRS-III, DBT, FAB, HADS, Tinetti mobility test, VFT, FoG-Q and TUG.	Structural DTI Functional Connectivity (FC) ROI seed-based analyses	Cross-sectional	Diffuse WM deficits in tracts projecting from PPN to motor, sensory & cognitive areas (C/T HC). Lower FC of right PPN with right MTG, ITG, & bilateral CRB (C/T to HC & FoG-)	Interference AND/OR Perceptual Dysfunction
Wang et al. (2021)	25 FoG+ 25 FoG- 25 HC	OFF	H&Y, LEDD, MMSE, UPDRS-III, new FoG-Q and clockwise vs anti-clockwise turning.	Functional connectivity (FC) ROI seed-based analyses	Cross-sectional Correlational (indirect with FoG-Q scores)	Higher dynamic FC between left thalamic nuclei and right IPL (positively correlated with *N*-FoGQ) & left PCG (C/T FoG- & HC).	Interference
Yu et al. (2021)	20 FoG+ 21 FoG- 18 HC	OFF	H&Y, LEDD, MMSE, UPDRS-III, CDT, DOT, FAB, HADS, MoCA, TMT, FoG-Q and TUG. All FoG+ are ‘OFF-FoG’ since they experienced FoG only during ‘OFF’ medication.	Functional Connectivity (FC) ICA to examine FC between dorsal attention network (DAN) and seven other RSNs.	Cross-sectional Correlational (indirect with FAB, CDT and TUG scores)	Lower positive internetwork FC between DAN and medial VN & SMN (C/T FoG- & HC) and between medial VN & SMN (C/T FoG-).Lower FC in medial VN, lateral VN & SMN and higher FC in DMN (C/T FoG- & HC)	Perceptual Dysfunction

Table 1: Summary of the methods and findings of the reported structural and resting-state functional connectivity studies investigating freezing of gait (FoG) in Parkinson’s disease (PD) patients.

ACE-R: Addenbrooke’s cognitive examination revised, AIT: alternating intake test, ANT: alternating names test, BADA: battery of assessment of aphasia disorders, BBS: Berg balance scale, BDI: Beck depression inventory, BG: basal ganglia, C/T: compared to, CA: constructional apraxia test, CC: corpus collosum, CCSIT: cross-cultural smell identification test, CDT: clock drawing test, CLR: cerebellar locomotor region, CRB: cerebellum, DBT: digit backward test, DMN: default mode network, DOT: digit ordering task, DTI: diffusion tensor imaging, FAB: frontal assessment battery, FoG+: PD patients with freezing of gait, FoG-: PD patients without FoG, FoG-Q: freezing of gait questionnaire, GP: globus pallidus, HADS: Hamilton anxiety and depression scale, HAM-D: Hamilton depression scale, HC: healthy controls, H&Y: Hoehn & Yahr, ICA: independent component analysis, ITG: inferior temporal gyrus, LEDD: levodopa equivalent daily dose, M1: primary motor cortex, MCI: mild cognitive impairment, MLR: mesencephalic locomotor region, m/lVN: medial/lateral visual network, MMSE: mini mental state exam, MoCA: Montreal cognitive assessment, MOG: middle occipital gyrus, MTG: middle temporal gyrus, PANDA: Parkinson’s neuropsychometric dementia assessment, PCG: postcentral gyrus, PDQ-39: Parkinson’s disease questionnaire [item assessing quality of life], PPT: pedunculopontine nucleus, RAVLT: Ray auditory verbal learning test, RCPM: Raven’s 47 colored progressive matrices, ROI: region of interest, RSNs: resting state networks, SMA: supplementary motor area, SMG: supramarginal gyrus, SMN: sensorimotor network, SPL: superior parietal lobule, STL: superior temporal lobe, STN: subthalamic nucleus, TMT: Trail making test, TPCT: ten point clock test, TUG: timed up and go, UPDRS: Unified Parkinson’s disease rating scale, VFT: verbal fluency test, WM: white matter.

### 3.2 Imaging studies of resting-state functional connectivity associated with FoG in Parkinson’s disease

A promising approach to investigate the neural mechanisms underlying FoG in PD patients is resting-state fMRI (rs-fMRI). This neuroimaging approach has been extensively used in studying the neural correlates of FoG in patients with PD as it permits the assessment of the whole brain without an experimental task. Rs-fMRI quantifies the functional connectivity between spatially disparate neural networks (intrinsic connectivity networks, ICN) by detecting fluctuations in spontaneous BOLD signals across the whole brain (Mancini et al., 2017). The parameters derived from rs-fMRI indicate a correlation (coupled) or an anti-correlation (anti-coupled) in the activity of the examined specific functional neural networks/ICNs (Biswal et al., 1995, Grayson and Fair, 2017).

Several rs-fMRI studies reveal a reorganization of the intrinsic functional connectivity (FC) within higher cortical regions (Droby et al., 2021) and subcortical regions such as the locomotor regions (Bharti et al., 2020, Wang et al., 2016) and the BG (Biswal et al., 1995, Vervoort et al., 2016) in PD patients with FoG. Notably, a higher FC between the CLR and other cerebellar areas positively correlated with FoG severity (Bharti et al., 2019a). Other rs-fMRI studies found abnormal connectivity patterns of cortical areas with subcortical structures in PD patients with FoG. In comparison to PD patients without FoG, PD patients with FoG showed an increase in the FC between mesencephalic locomotor regions and cortical sensorimotor areas (Fling et al., 2014, Lench et al., 2020), putamen and precuneus (Vervoort et al., 2016), GP and primary somatosensory cortex (Miranda-Domínguez et al., 2020), thalamus and right inferior parietal lobule (Wang et al., 2021), as well as between the ventral tegmental area and areas of the default mode network (Steidel et al., 2021). Notably, all these abnormalities in functional connectivity between cortical and subcortical structures were shown to be positively correlated to behavioral or subjective measures of FoG. Increased functional connectivity (FC) between cortical and subcortical structures may indicate a maladaptive compensatory strategy for the lack of automaticity in PD patients with FoG (Wu et al., 2015). These findings align with the ‘interference’ model of FoG, positing that more robust maladaptive recruitment of cortical areas and impaired crosstalk between cortical and subcortical areas lead to a cognitive “overload” of the striatum that in turn causes FoG.

It is well established that PD patients, and those suffering from FoG in particular (Klucken et al., 2015), rely more on higher-order brain areas to control gait under the circumstances in which gait is usually controlled automatically, e.g., simple walking or turning (Wu et al., 2015). However, the observation of a positive correlation between objective measures of freezing and a higher FC between the SMA and MLR (Fling et al., 2014) as well as between the GP and the primary somatosensory cortex (Miranda-Domínguez et al., 2020) suggests that a higher-order control of gait is not always compensatory in patients with FoG. It is not clear whether the observed lack of compensatory recruitment of cortical areas for gait control in PD patients with FoG is the result of a primary (or initial) failure in the implementation of higher-order control of gait in PD patients with FoG or the result of a secondary deterioration in the efficiency of higher-order gait control when the disease progresses. A potential answer can be derived from a longitudinal experimental design recently adopted by Jung and colleagues (2020) that compared resting-state FC acquired at baseline in PD patients who later developed (FoG-vulnerable group) or did not develop (FoG-resistant group) FoG within 5 years from scanning. At baseline, the authors found a comparable FC between cerebellar structures and the SMA in both patient groups suggesting the abnormal increase in FC of subcortical areas with the SMA in patients with FoG (observed in other studies) is likely a phenomenon that emerges concurrently with FoG.

Dysfunctional network connectivity between subcortical and cortical areas extended beyond cognitive and sensorimotor areas, also affecting visuospatial networks. In PD patients with FoG, abnormal FC was observed between the visual cortex, mainly of the right hemisphere, and left caudate (Steidel et al., 2021), right PPN (Wang et al., 2016), bilateral thalamus and GPe (Potvin-Desrochers et al., 2019), when compared to PD patients without FoG. Moreover, two studies found distinct FC alterations between cerebellar and parieto-occipital regions. While one study detected an increased FC between the cerebellum and parieto-occipital regions in PD patients with FoG (Bharti et al., 2019a), another study observed a decrease in FC between these areas in PD patients vulnerable to developing FoG (Jung et al., 2020). Given differences in the studied samples and experimental designs, these disparate findings are not necessarily contradictory but can also be considered complementary. One plausible interpretation to account for the different FC patterns is that a functional decoupling between the cerebellum and parieto-occipital areas is a potential biomarker leading to or preceding the emergence of FoG symptoms, which once developed, can produce an opposite trend of an abnormally high FC between these two areas. A systematic investigation that directly compares resting-state FC of PD patients before the occurrence of FoG symptoms with their resting-state FC following the onset of FoG is warranted to understand the pathophysiology of FoG in PD better.

Several studies converge in reporting a decrease in resting-state FC between several cortical areas along the dorsal stream of visuomotor processing (Canu et al., 2015, Lenka et al., 2016, Miranda-Domínguez et al., 2020, Yu et al., 2021). In particular, PD patients suffering from FoG exhibited lower FC between the visual and sensorimotor networks than PD patients without FoG (Yu et al., 2021). Moreover, the severity of FoG was negatively correlated with the FC within the right visual network (Tessitore et al., 2012). Together, the revealed decoupling in resting-state FC within occipitotemporal networks and the previously discussed abnormal FC of visual networks with subcortical structures support the crucial role of perceptual dysfunction in the emergence of FoG in PD patients. Importantly, these abnormalities were mainly observed in the right hemisphere, which coincides with the more pronounced right-lateralized WM structural abnormalities in PD patients with FoG (Canu et al., 2015, Fling et al., 2014, Fling et al., 2013, Pietracupa et al., 2018). The right hemispheric lateralization of abnormalities in structural and functional connectivity further supports the contribution of visuospatial impairments to the genesis of FoG. It is well established that the right hemisphere is predominantly responsible for spatial cognition and visuospatial processing that are, in turn, essential for efficient spatial navigation. Given the observed right-hemispheric occipitotemporal dysfunction in PD patients with FoG, these patients might have additional difficulties in processing and integrating visuospatial information during the motor planning of gait, which renders this population at a higher risk of experiencing an episode of FoG in novel or complex environments, such as passing through narrow doorways. For instance, PD patients with FoG showed more robust connectivity of the putamen with the retrosplenial cortex, an area linked to spatial cognition and locomotion in space (Mitchell et al., 2019). This finding suggests an enhanced dependence on external sensory cues to compensate for deficient visuospatial processing in PD patients with FoG (Potvin-Desrochers et al., 2019).

The significant findings derived from resting-state fMRI (rs-fMRI) studies primarily support the ‘interference’ or the ‘perceptual dysfunction’ models of the pathophysiology of FoG in PD. These findings should nevertheless be treated with caution, given several inherent limitations of the rs-fMRI method in FoG in PD. On the one hand, rs-fMRI studies of FoG mainly adopt a cross-sectional approach that compares the neurophysiological changes of PD patients with FoG with those of healthy controls or PD patients without FoG at a given time. A fundamental shortcoming of such a cross-sectional approach is the failure to consider the contribution of interindividual variability in FoG severity within groups of PD patients with FoG. In particular, the sole comparison of resting-state FC changes in PD patients with FoG versus healthy controls can be even more problematic than the comparison of PD patients with FoG with PD patients without FoG, as the former comparison cannot differentiate between specific neuroanatomical/-physiological changes related to FoG and those related to other more general deficits (clinical, motor or cognitive) in Parkinson’s disease. To circumvent these limitations of cross-sectional approaches, the alternative study design adopted by Jung et al. (2020, comparing baseline resting-state FC of patients that later did develop or did not develop FoG within 5 years from scanning) is a valuable approach to identify neurophysiological biomarkers predicting the likelihood and latency of FoG occurrence in PD patients. However, their design could even be improved by longitudinal rs-fMRI measurements (in addition to the baseline rs-fMRI) in groups of PD patients with different degrees of FoG. Such longitudinal designs may also reduce the putative effects of confounding factors in studies assessing specific FoG-related structural and functional brain changes since a given PD patient may serve as her/his control (e.g., before and after developing FoG).

On the other hand, rs-fMRI studies can only indirectly correlate resting state FC to FoG measures, as these studies relate neurophysiological changes measured when lying supine in the MRI scanner to objective or subjective gait assessments obtained outside the scanner. Except a few studies that related resting-state FC changes with objective measures of FoG recorded during a turning or walking task before the scanning session, most of the rs-fMRI studies on FoG in PD related resting-state FC changes with subjective self-reports of FoG, i.e., using the FoG-Questionnaire. However, the reliability of subjective recall of FoG symptoms may be hampered by cognitive deficits. An alternative objective assessment tool of FoG that can be considered in future rs-fMRI studies is the rating instrument developed by Ziegler et al., (2010), which generates a ‘FoG score’ that quantifies the patient’s performance across different motor tasks that incorporate common triggers of FoG (e.g., turning).

## 4 Task-based imaging studies of FoG in Parkinson’s disease

Task-based fMRI studies can detect real-time correlations of neurophysiological alterations with gait-related performance measured during the scanning session. These studies mainly adopt virtual reality (VR) and motor imagery (MI) paradigms that correlate neurophysiological changes with foot-pedaling performance and with the mental simulation of pre-instructed (gait-related) actions, respectively. In addition to VR and MI paradigms, a recent study adopted a novel paradigm of dorsal or plantar foot-flexion movement in response to auditory stimuli, which meant to approximate the sensorimotor activity during normal gait without inducing episodes of FoG (Piramide et al., 2020). Importantly, these authors divided PD patients with FoG into two subgroups of differing FoG severity: PD patients with mild FoG and PD patients with moderate FoG. The comparison of neurophysiological changes between patient groups with different degrees of FoG severity (as assessed by the FoG-Questionnaire) can be more insightful than the comparison of PD patients with FoG and without FoG, mainly since the former comparison permits the detection of distinct alterations corresponding to different levels of FoG severity. Compared to PD patients with mild FoG, patients with moderate FoG showed reduced activity in frontoparietal regions and the frontostriatal circuits, paralleled by increased compensatory recruitment of parieto-occipital regions, all of these observations were positively correlated with FoG severity. These findings suggest a switch in the adopted compensatory strategies in PD patients with FoG along with the progression of their FoG symptoms severity. In mild stages of FoG, PD patients attempt to compensate for their gait impairments via higher-level cognitive control strategies, while they instead adopt visuospatial strategies in more severe stages of FoG.

### 4.1 Task-based MRI studies employing virtual reality (VR) paradigms

In combination with functional neuroimaging, virtual reality (VR) paradigms were used in several studies to explore the underlying pathophysiology of FoG in PD patients. Virtual reality is supposed to permit an objective and safe evaluation of FoG in PD patients in an ecologically valid environment. The reported VR paradigms consist of first-person perspective navigation. Participants navigate using MRI-compatible foot pedals through a virtual environment that displays several triggers known to produce FoG in PD patients (e.g., narrow doorways or turning initiation). In these studies, FoG is measured as an episode of motor arrest, consistently defined across studies as an abnormally long period of between-footstep latency compared to the mean between-footstep latency computed during normal walking. Measures of these motor arrests were positively correlated with self-reported severity of FoG (Naismith and Lewis, 2010, Shine et al., 2011), suggesting that motor arrests during VR in fMRI can be used as a surrogate marker for actual episodes of FoG.

Shine and colleagues conducted a series of VR studies revealing consistent alterations in BOLD activation levels and functional connectivity (FC) changes between cortical and subcortical regions of PD patients with FoG. Increased activation in frontoparietal regions was negatively related to freezing severity, whereas reduced activity in the BG, thalamus (Shine et al., 2013a) and pre-SMA (Shine et al., 2013c) were observed during episodes of motor arrests. In a follow-up study with a separate cohort, task-based FC analyses revealed a functional decoupling between the cortical cognitive control network (including the dorsolateral prefrontal cortex and posterior parietal cortex) and the subcortical BG network during episodes of motor arrests in PD patients with FoG, particularly in the OFF dopaminergic state relative to the ON dopaminergic state (Shine et al., 2013b). These findings suggest that episodes of motor arrests (representing FoG) are manifestations of impaired activation in and connectivity between frontoparietal neuronal networks responsible for goal-directed action and subcortical networks responsible for automatic gait control. During gait with a concurrent cognitive task, an overload of the frontostriatal network leads to a breakdown in gait functions expressed by a reduced inhibitory influence of the cognitive control network onto the BG’s output, in turn resulting in an (over-)inhibition of the MLR/PPN (Lewis and Barker, 2009). The pathological inhibition of these locomotor regions is considered a potential mechanism triggering FoG in PD.

In agreement with the findings reported by Shine and colleagues, further VR studies revealed abnormal functional connectivity (FC) patterns between cortical and subcortical structures (Ehgoetz Martens et al., 2018, Matar et al., 2019). Notably, a decoupling between the cognitive control network and the striatum (Ehgoetz Martens et al., 2018) was associated with a reduced FoG severity, while a decoupling between the pre-SMA and the STN (Matar et al., 2019) led to an increased FoG severity. In addition, abnormally increased FC within several subcortical structures has been reported (Ehgoetz Martens et al., 2018, Gilat et al., 2015, Matar et al., 2019). Relative to PD patients without FoG, PD patients with FoG exhibited higher FC between the GPi and the STN as well as the MLR (Gilat et al., 2015) and between the STN bilaterally (Matar et al., 2019). Moreover, dysfunctional activation patterns were observed in higher-level cortical structures, including hypoactivation in bilateral pre-SMA (Matar et al., 2019) and left premotor area (Gilat et al., 2015) in PD patients with FoG.

Most of the above-reported abnormalities concentrate on the ‘hyper-direct’ pathway (see Fig. 1), comprising the pre-SMA and the STN, which allows the premotor regions to directly affect motor output by projecting directly to the STN, thus bypassing the striatum (Nambu et al., 2002). Note that this ‘hyper-direct’ pathway has also been implicated in response inhibition and conflict resolution (Cavanagh and Frank, 2013). Therefore, the observed functional decoupling between the pre-SMA and the STN, along with the reduced activation in the former (Matar et al., 2019), would lead to impairments in resolving conflicts between competing yet complementary motor programs (Lewis and Shine, 2016a). The failure in selecting the proper motor response would produce an excessive excitatory output from the STN to the GPi, as evidenced by a higher coupling between the two (Gilat et al., 2015), which, in turn, will increase the GABAergic inhibition of locomotor regions and eventually manifest into an episode of FoG (Lewis and Barker, 2009).

Despite differences in the implemented VR experimental paradigms, most of the neuroimaging findings revealed by task-based VR paradigms support the predictions made by the 'interference model' (Lewis and Barker, 2009, Lewis and Shine, 2016). Taken together, findings from VR studies reveal a common neural pathway that underlies all clinical manifestations of FoG, namely, a dysfunctional increase in inhibitory efferent output from the BG output nuclei (GPi and SNr) to the brainstem structures that control locomotion (MLR/PPN) and to the motor thalamus (see Fig. 2). In particular, the hyperactivity in the GPi can result from multiple types of competing cognitive, motor, or limbic processes that are inefficiently mediated or resolved at the level of the ‘hyper-direct’ pathway. Consequently, the dysfunction in cortico-striatal networks leads to an excessive inhibitory outflow from the BG output nuclei either by a hyperactivation of excitatory efferents from the STN to the GPi or by a hypo-activation of inhibitory efferents from the striatum to the GPi (see Fig. 1, Fig. 2). The above-summarized fMRI studies employing task-based VR paradigms in PD patients with FoG demonstrate both types of failure in the neural control of gait, i.e., FoG was shown to be associated with hyperactivity in the STN firing rate toward the GPi (Gilat et al., 2015, Matar et al., 2019) and hypoactivity in the firing rate of the striatum toward the GPi (Ehgoetz Martens et al., 2018, Shine et al., 2013a).

**Table 2:** Task-based MRI studies assessing freezing of gait (FoG) in Parkinson’s disease (PD) patients.

Author/Year	Sample	Dopaminergic Status	Clinical/neuropsychological parameters and FoG/gait assessment	Imaging Modality and Parameters	Experimental design	Main Findings	Supported Model(s)
Crémers et al. (2012)	15 PD 15 HC	ON	MoCA, TMT, VMIQ-2, and 25 m walking test	Activation (BOLD)	MI paradigm: gait imagery of walking vs standing. Cross-sectional Correlational (direct with MI scores)	MI of walking: lower activations in the PPN/MLR, CRB, parieto-occipital regions, & left hippocampus (C/T HC). Higher activity in PD in right posterior parietal cortex & pre-SMA negatively correlated with gait severity (25-m task).	Perceptual Dysfunction
Ehgoetz Martens et al. (2018)	20 FoG+	OFF	MMSE, UPDRS-III, HADS, TMT, and FoG-Q	Functional connectivity (FC)	VR paradigm: walking with foot pedals (high vs low cognitive load). Cross-sectional Correlational (direct with motor arrests, indirect with cognitive tests)	Lower FC between the striatum and the CCN & the motor network, and higher FC between the putamen & striatum (during motor arrests)	Interference
Gilat et al. (2015)	17 FoG+ 10 FoG-	OFF	H&Y, LEDD, UPDRS, FoG-Q and III, TUG. Lower cognitive performance (MoCa) and higher self-reports of depression and anxiety (HADS controlled for).	Activation (BOLD) Functional connectivity (FC)	VR paradigm: straight walking vs turning with foot pedals (with & without a cognitive task). Cross-sectional Correlational (direct with motor arrests)	Turning: lower BOLD in left premotor area & left SPL, and higher BOLD in bilateral IFG (C/T FoG-). Higher FC in FoG + between bilateral MLR, left GPi & bilateral MLR, and right GPi & left STN (C/T walking)	Interference
Huang et al., (2021)	22 FoG+ 15 FoG- 15 HC	OFF	H&Y, LEDD, MMSE, PDQ-39, UPDRS-III, VMIQ, new FoG-Q and 50 m walking test.	Activation (BOLD) ROI (MLR, CLR, SLR)	MI paradigm: first person video-guided gait imagery of normal gait (walking and 360 degrees turning) vs FoG gait (FoG walking and FoG turning). Cross-sectional Correlational (direct with MI)	Normal turning: higher activations in FoG+ in locomotor regions (MLR, CLR and SLR) C/T FoG-. FoG turning: higher activation in pre-& post-central gyrus & superior occipital lobule (C/T FoG- & FoG walking) .	Perceptual Dysfunction
Matar et al. (2019)	19 FoG+	ON and OFF	FoG-Q Longer footsteps latencies in the OFF vs ON state.	Activation (BOLD) Functional connectivity (FC)	VR paradigm: navigating through wide or narrow doorways with foot pedals. Cross-sectional Correlational (direct with motor arrests) Comparative (medication state)	OFF-state: Longer footstep latencies positively correlate with hypoactivation in bilateral pre-SMA, and lower FC of pre-SMA & STN bilaterally. Frequency of longer footstep latencies correlated with higher FC between the bilateral STN	Interference
Peterson et al. (2014a)	9 FoG+ 9 FoG-	OFF	H&Y, MMSE, UPDRS-III, GIQ, KVIQ and new FoG-Q	Activation (BOLD) ROI	Motor imagery (MI): gait imagery of standing, turning, forward or backward walking. Cross-sectional Correlational (direct with MI, indirect with FoG-Q and gait task)	Lower BOLD in right GP during all MI tasks, and lower BOLD in CLR during imagined stand, and a trend to lower BOLD in right SMA & MLR (C/T FoG-)	Interference
Peterson et al. (2014b)	19 PD (9 FoG+) 20 HC	OFF	MMSE, GIQ, KVIQ and new FoG-Q	Activation (BOLD) ROI	MI paradigm: gait imagery of standing, right or left turning, and backward or forward walking. Cross-sectional Correlational (direct with MI, indirect with gait task)	Lower activation (C/T HC) in left GP in all tasks and a higher activation in right SMA during turning (C/T walking). Higher activation of SMA & GP in PD positively correlated with gait velocity	Interference
Piramide et al. (2020)	17 FoG+ (9 mild and 8 moderate FoG+) 10 FoG- 18 HC *Note*: 15 patients had MCI (12 FoG+, 3 FoG-).	OFF	MMSE, PDQ-39, tests for executive functions, visuospatial skills, memory & language. FoG-Q (Mild = median value ≤ 11; Moderate = median value > 11), TUG, and 10 m walking test. FoG+ had worse scores on executive and language tests	Activation (BOLD)	Foot flexion task. Cross-sectional Correlational (direct with gait measures of dorsal and plantar foot flexion and indirect with clinical measures)	Lower activity of fronto-parietal areas in moderate FoG+ (C/T mild FoG+). FoG severity correlated with higher activation in parieto-occipital areas and reduced activation of fronto-striatal circuit & fronto-parietal areas. Lower activity of right putamen correlate with worse executive attention/WM & visuospatial functions in FoG+	Interference AND/OR Perceptual Dysfunction
Shine et al. (2013a)	18 FoG+	OFF	FoG-Q and TUG	Activation (BOLD)	Virtual Reality (VR) paradigm: walking with MRI compatible foot pedals (high vs low cognitive load conditions). Correlational (direct with motor arrests, indirect with FoG-Q scores) .	Lower BOLD in sensorimotor cortex and higher BOLD in fronto-parietal regions, negatively correlated with FoG-Q. Lower BOLD in MLR positively correlated with FoG-Q, and lower BOLD in GPi, caudate & thalamus correlated with motor arrests	Interference
Shine et al. (2013b)	10 FoG+ 10 FoG-	ON and OFF	H&Y, MMSE, UPDRS-III, FoG-Q and TUG. More motor arrests in FoG+ during low and high cognitive load conditions	Activation Functional connectivity (FC) ICA	VR paradigm: walking with foot pedals (high vs low cognitive load). Cross-sectional Correlational (direct with motor arrests) Comparative (medication state)	Motor arrests correlate with a functional decoupling between BG network & bilateral CCN in FoG+. Impaired cross talk between left & right CCN in FoG+ (during high cognitive load)	Interference
Shine et al. (2013c)	14 FoG+ 15 FoG-	OFF	HADS, MoCa, FoG-Q and TUG FoG+: higher self-reports of anxiety and depression	Activation (BOLD)	VR paradigm: walking with foot pedals (high vs low cognitive load). Cross-sectional Correlational (direct with motor arrests)	High cognitive-load task: lower BOLD in anterior insula bilaterally, pre-SMA, left STN & ventral striatum bilaterally (C/T FoG-)	Interference
Snijders et al. (2011)	12 FoG+ 12 FoG- 21 HC	OFF	MMSE, UPDRS, FAB, VMIQ-2 and new FOG-Q	Activation	MI paradigm: gait imagery of walking along a path of different widths. Cross-sectional Correlational (direct with MI, indirect with FoG-Q)	Higher activity in MLR during MI of gait, and lower activity in mesial frontal & posterior parietal regions (C/T FoG-).	Interference
Wai et al. (2012)	13 PD 13 older HC 14 young HC	Not specified	H&Y and UPDRS-III	Activation (BOLD)	MI paradigm: gait imagery of initiation, termination and stepping over obstacles. Cross-sectional Correlational (direct with MI)	Stepping over obstacles: Higher activation in right dorsal premotor area, right IPL, & bilateral precuneus (C/T HC-old)	Perceptual Dysfunction

Table 2: Summary of the methods and findings of the reported task-based MRI studies assessing freezing of gait (FoG) in Parkinson’s disease (PD) patients.

BG: basal ganglia, C/T: compared to, CCN: cognitive control network, CLR: cerebellar locomotor region, DBS: deep brain stimulation, FAB: frontal assessment battery, FoG+: PD patients with freezing of gait, PDQ-39: Parkinson’s disease questionnaire [item assessing quality of life], FoG-: PD patients without FoG, FoG-Q: freezing of gait questionnaire, GFQ: gait and fall questionnaire, GIQ: gait imagery quotient, GPi: globus pallidus internus, HADS: Hamilton anxiety and depression scale, HC: healthy controls, H&Y: Hoehn & Yahr, IFG: inferior frontal gyrus, IPL: inferior parietal lobe, KVIQ: kinesthetic visual imagery quotient, LEDD: levodopa equivalent daily dose, MI: motor imagery, MIQ-R: revised movement imagery questionnaire, MLR: mesencephalic locomotor region, MoCA: Montreal cognitive assessment, MMSE: mini mental state exam, PDQ-39: Parkinson’s disease questionnaire, PPN: pedunculopontine nucleus, rCBF: regional cerebral blood flow, ROI: region of interest, SMA: supplementary motor area, SPL: superior parietal lobe, STN: subthalamic nucleus, TMT: Tinetti mobility test, TUG: timed up and go, UPDRS: Unified Parkinson’s disease rating scale, VMIQ-2: vividness of movement imagery, VR: virtual reality, WM: working memory.

### 4.2 Task-based MRI studies employing motor imagery (MI) paradigms

Motor imagery (MI) paradigms are adopted to assess and modulate gait impairments in PD patients. MI is defined as the mental simulation of action without overt execution (Jeannerod, 1994). MI simulates complex cognitive and perceptual processes related to motor cognition. For instance, imagined gait movements similarly obey Fitts’ laws as actual movements (Decety and Jeannerod, 1995). In gait control, MI allows examining the ‘feedforward’ mechanisms that incorporate sensory and motor predictions of an action without influencing ‘feedback’ mechanisms (de Lange et al., 2005, Lotze and Halsband, 2006). This sets MI apart from the VR approach (Mirelman et al., 2013). MI helps assessing the neural mechanisms that underlie gait control in healthy people and patients since imagining a specific movement produces similar brain activation patterns as its actual execution (Stephan et al., 1995).

Following the reported hypoactivation in subcortical regions in VR studies (Shine et al., 2013a, Shine et al., 2013c), imaging studies employing MI paradigms in PD patients with gait impairments revealed altered activation patterns in several subcortical regions, mainly in the GP (Peterson et al., 2014a, Peterson et al., 2014b) and in the locomotor regions (Crémers et al., 2012, Peterson et al., 2014a). In particular, reduced activity in the CLR and the right GP was detected during imagined gait in PD patients with FoG than PD patients without FoG (Peterson et al., 2014a). In contrast, other MI studies comparing imagined gait between PD patients with FoG and without FoG reported abnormal activity increases in several locomotor regions (Huang et al., 2021) that positively correlated with disease duration in the PD patients with FoG. Moreover, increased activation in the MLR was positively correlated with FoG severity (Snijders et al., 2011). Notably, another MI study investigating gait improvements in PD patients following subthalamic deep brain stimulation (STN-DBS), reported an association between higher MLR/PPN activation and gait improvements when STN-DBS was switched on compared to STN-DBS off (Weiss et al., 2015). The latter finding suggests a compensatory role of the increased activity in subcortical structures for overcoming gait deficits in PD, which may extend to overcoming deficits related to FoG in PD. Thus, the observed discrepancies in MI studies concerning increased versus decreased activation of subcortical regions may be related to the clinical status of the investigated sample of PD patients with FoG, as PD patient with FoG in more advanced stages of the disease require a more robust activation of subcortical structures to overcome episodes of FoG.

Several MI studies revealed abnormal patterns of activation encompassing cortical areas along the dorsal visuomotor pathway that were more pronounced in the right hemisphere of PD patients with gait impairments (Crémers et al., 2012, Wai et al., 2012). During MI of a FoG-inducing turning task, PD patients with FoG exhibited increased recruitment of parieto-occipital regions relative to PD patients without FoG (Huang et al., 2021). The recruitment of visuospatial cortical areas by PD patients during MI is particularly remarkable given that most MI paradigms did not include external visual input or feedback to guide MI of gait. Note that, in a sample of healthy older participants, an increase in the activation of temporal and parietal regions was only observed when visual cues were provided during a MI task compared to MI without visual cues (Zapparoli et al., 2020). Together these observations further corroborate the notion that PD patients, especially those with FoG, heavily rely upon visual strategies and visuomotor coordination to guide their gait.

Studies adopting MI paradigms report various neurophysiological changes that primarily show agreement with either the ‘interference’ or the ‘perceptual dysfunction’ models of FoG in PD. The variability in observed findings across MI studies is partly due to the differences in complexity across the implemented MI paradigms and differences in the studied samples of PD patients. The latter is particularly problematic for a cross-studies comparison since the included patients show a wide range of disease severity. Moreover, the lack of FoG assessments in some MI studies renders it difficult to precisely distinguish between neurophysiological changes associated with general symptoms of PD and those mainly linked to FoG in PD.

### 4.3 Activation studies of actual gait employing functional near-infrared spectroscopy (fNIRS)

Functional near-infrared spectroscopy (fNIRS) is a neuroimaging method that recently gained popularity in studying the neurophysiology of gait impairments and FoG in PD. Like fMRI, this technique detects hemodynamic changes (of oxygenated and deoxygenated hemoglobin) in cerebral blood flow produced by neurovascular coupling. Being both portable and wireless, fNIRS permits measurements of brain activity during actual walking, i.e., while being in an upright position, thus having the advantage of also assessing the postural and balance control mechanisms involved in bipedal gait (in contrast to the supine position when lying in an MRI scanner). Moreover, fNIRS studies allow the direct correlation of neurophysiological changes with real-time behavioral gait metrics measured by wearable gait sensors. Nieuwhof and colleagues (2016) have demonstrated the feasibility of using portable fNIRS to measure the brain activity of PD patients, in particular, activity within the prefrontal cortex (PFC), during different conditions of dual-task walking. Consecutive fNIRS studies investigated gait impairments and their association with changes in PFC activity in PD patients under different experimental paradigms and treatment conditions.

Studies investigating prefrontal activity with fNIRS during a walking task converge on reporting higher activity within the PFC (Maidan et al., 2016, Nieuwhof et al., 2016) and the DLPFC (Ranchet et al., 2020) in PD patients, mainly in comparison to healthy control participants. These observations agree with the notion of compensatory recruitment of executive control functions during the execution of basic motor operations in PD patients with prevalent deficits in gait automaticity (Wu et al., 2015). The switch from automatic to voluntary higher-level gait control appears to be directly associated with gait impairments and disease severity in PD patients. In particular, PD patients with the more severe and FoG-related postural instability and gait disorder (PIGD) subtype demonstrated an enhanced reliance on executive functions during normal gait in comparison to PD patients with the less severe tremor dominant (TD) subtype (Orcioli-Silva et al., 2020). In parallel to these observations, fNIRS studies that compared PFC activity between PD patients with and without FoG revealed a higher PFC activation during single and dual task walking (Vitorio et al., 2020) as well as during single task turning (Belluscio et al., 2019) that positively correlated with worse FoG measures in PD patients with FoG.

Furthermore, studies assessing the effect of dopamine treatment on the recruitment of executive functions during gait indicate that PD patients with FoG exclusively employ executive functions to alleviate the loss of movement automaticity, as evidenced by increased recruitment of the PFC during gait (Dagan et al., 2020). In contrast, PD patients without FoG seem to adopt other strategies to successfully compensate for the reduced movement automaticity in PD, as they showed even a decrease in PFC recruitment (Stuart et al., 2020). Note that the discrepancy in the adopted compensatory strategy between PD patients with and without FoG can also be partially accounted for by the duration of levodopa-replacement therapy. In PD patients without FoG and early stages of PD, dopaminergic treatment can be efficient in modulating the recruitment of prefrontal areas to alleviate gait deficits without simultaneously overloading executive and attentional resources. However, PD patients with FoG and in advanced stages of PD may develop a pseudo-resistance to levodopa treatment that impedes its treatment efficacy (Kouli et al., 2018). A thorough understanding of the relationship between dopaminergic treatment and cognitive load in PD patients with FoG warrants future (imaging) studies that directly compare PD patients with different disease severity during both ON– and OFF- medication conditions and preferably include disease duration as a covariate in the analysis of the imaging data.

Since all fNIRS studies to date were restricted to the investigation of frontal brain regions associated with executive functions, i.e., the PFC and the DLPFC, their findings are largely discussed in light of the ‘executive dysfunction’ model of FoG in PD. Nevertheless, some fNIRS results indicate a potential reliance on other strategies, e.g., visuospatial strategies, to compensate for the loss of gait automaticity in PD patients with FoG. Given that dual-task turning requires higher cognitive control than single-task turning and, consequently, higher activation of the PFC, the counterintuitive observation of lower recruitment of PFC in PD patients suffering from FoG during dual-task turning (Belluscio et al., 2019) suggests that these patients relied on other higher-level areas when faced with challenging gait control conditions. In line with the findings by Piramide et al. (2020), PD patients with FoG might have employed visuospatial strategies to regulate gait during difficult conditions if compensation via executive control strategies was inefficient. This is further corroborated by the reported deficits in executive functions in the studied sample of PD patients with FoG and the positive correlation of their PFC activity with FoG severity and visuospatial impairments (Belluscio et al., 2019). Moreover, the implementation of external visuospatial cues helped the PD patients to adaptively modulate their gait without burdening executive control resources, as evidenced by reduced PFC activation in PD patients during treadmill walking, which affects gait rhythmicity, as compared to over-ground walking (Thumm et al., 2018). Accordingly, external visuospatial cues can be used to compensate for visuospatial processing deficits in PD patients, especially in complex or novel environments. Note that a recent study (Bonnet et al., 2020) reported reduced automatic visuomotor coupling and increased attentional demands to maintain standard posture in the presence of distracting stimuli, which was shown to induce episodes of falling or FoG in PD patients.

**Table 3:** Activation studies investigating actual gait in PD patients employing functional near-infrared spectroscopy (fNIRS).

Author/Year	Sample	Dopaminergic Status	Clinical/neuropsychological parameters and FoG/gait assessment	Experimental Design	Main Findings	Supported Model(s)
Belluscio et al. (2019)	15 FoG+ 17 FoG- 8 HC	OFF	UPDRS, CLOX, FAB, MoCa, TMT, and new FoG-Q FoG- had worse MoCa scores	Single vs dual task turning in place with wearable inertial sensors. Cross-sectional Correlational (direct with real-time gait measures and indirect with NFoG-Q & visuospatial scores)	Activation of the PFC: higher in single turning, and lower in dual-task turning (C/T FoG- & HC). Single turning: Higher PFC activation in FoG+ is linked to higher FoG ratio & worse visuospatial abilities	Executive Dysfunction AND/OR Perceptual Dysfunction
Dagan et al. (2020)	40 FoG+	ON and OFF	LEDD, MMSE, UPDRS-III, global cognitive tests, and new FoG-Q	Single vs dual task walking with wearable inertial sensors. Comparative (medication state) Correlational (direct with real-time gait measures)	Single task: higher PFC activation in the ON relative to the OFF condition. Dual task: no increase in PFC activation in the ON relative to the OFF condition	Executive Dysfunction
Maidan et al. (2016)	68 PD 38 (older) HC	ON	H&Y, MMSE, UPDRS, FSST, tests of executive functions, attention & visuospatial processing, and 2 min walking test. PD had lower scores on MMSE and all cognitive tests	Regular walking, dual task (DT) walking, and obstacle avoidance Cross-sectional Correlational (direct with real-time gait measures)	Higher PFC activation during usual walking (C/T HC) and during obstacle avoidance (C/T to usual walking). Higher PFC activation in PD was positively correlated with higher gait speed during OA, and lower PFC activity was correlated with more severe disease symptoms during usual walking & obstacle avoidance.	Executive Dysfunction
Nieuwhof et al. (2016)	12 PD	ON	H&Y, MMSE, PAQ, and FES-I	Walking while counting forward, serially subtracting, and reciting digit spans. Correlational (direct with real-time gait measures)	Higher activation in bilateral PFC during walking while subtracting and reciting digit spans compared to rest	Executive Dysfunction
Orcioli-Silva et al., (2020)	17 PD-PIGD 19 PD-TD	ON	MMSE, UPDRS, and TMT	Regular walking and obstacle avoidance Cross-sectional Correlational (direct with real-time gait measures)	PD-PIGD: higher bilateral PFC activity in all conditions (C/T PD-TD). A higher activation in right PFC in both groups during obstacle avoidance (C/T regular walking)	Executive Dysfunction
Ranchet et al. (2020)	18 PD 18 HC	ON	H&Y, LEDD, UPDRS-III, BDI, DSST, FES-I, MoCa, PMT, TMT, Stroop and Bells test. PD performed worse on the TMT, PMT, and Stroop word tasks	Standing while subtracting, regular and dual task walking (subtracting, counting forward) with wearable inertial sensors Cross-sectional Correlational (direct with real-time gait measures and indirect with TMT, DSST & Stroop test)	PD had higher DLPFC activity during regular walking and walking while subtracting. Higher DLPFC activity correlated with high gait variability during regular walking & walking while counting forward.	Executive Dysfunction
Stuart et al. (2020)	19 FoG-	OFF and ON– levodopa-placebo, or ON-levodopa-donepezil	H&Y, LEDD, MMSE, UPDRS-III, CLOX, CRT, FAB, MoCa, SRT, TMT, and FoG-Q (FoG+ were excluded).	Single and dual task walking. Comparative (medication state) Correlational (direct with real-time gait measures)	Lower PFC activity in ON-levodopa condition (C/T OFF). Higher PFC activity in ON-levodopa-donepezil (C/T OFF) correlated with better gait measures and accuracy during dual task	Executive Dysfunction
Thumm et al. (2018)	20 PD	ON	H&Y, LEDD, UPDRS-III and MoCa	Over-ground walking vs treadmill walking with wearable inertial sensors. Correlational (direct with real-time gait measures)	Treadmill walking was associated with lower activation in the PFC and a higher gait stability (C/T regular walking)	Perceptual Dysfunction
Vitorio et al. (2020)	24 FoG+ 23 FoG-	OFF	H&Y, UPDRS, CLOX, FAB, MoCa, TMT, and new FoG-Q. FoG+ scored worse in general cognition (controlled for)	Single and dual-task walking with portable gait sensors. Cross-sectional Correlational (direct with real-time gait measures and indirect with FoG-Q)	Single and dual-task: higher PFC activity in FoG+ (C/T FoG-). Dual-task: higher PFC activity was associated with less severe FoG & lower step-time variability in FoG+	Executive Dysfunction

Table 3: Summary of the methods and findings of the reported activation studies investigating actual gait in PD patients employing functional near-infrared spectroscopy (fNIRS).

Note that all the reported fNIRS studies measured changes in oxy-hemoglobin and deoxy-hemoglobin in the PFC or the DLPFC.

BDI: Beck depression inventory, C/T: compared to, CLOX: royal clock drawing task, CRT: choice reaction time, DLPFC: dorsolateral prefrontal cortex, DSST: digit symbol substitution test, FAB: frontal assessment battery, FES-I: Fall efficacy scale international, FoG+: PD patients with freezing of gait, FoG-: PD patients without FoG, FoG-Q: freezing of gait questionnaire, FSST: four square step test, HC: healthy controls, H&Y: Hoehn & Yahr, PAQ: physical activity questionnaire (version used by Amsterdam longitudinal aging study), LEDD: Levodopa equivalent daily dose, MMSE: mini mental state exam, MoCA: Montreal cognitive assessment, OA: obstacle avoidance, PD: Parkinson’s disease patients, PFC: prefrontal cortex, PIGD: postural instability gait disorder, PMT: plus-minus task, SRT: serial reaction time, TD: tremor dominant, TMT: Trail making test, UPDRS: Unified Parkinson’s disease rating scale.

## 5 Conclusion and outlook

The neuroimaging data associated with FoG in PD reveal that this phenomenon cannot be pinned down to a specific impairment in a single region within the gait control hierarchy. FoG is associated with dysfunction at diverse levels of the gait control system, including impaired communication and disturbed spatiotemporal dynamics between its components. Overall, neuroimaging findings from different modalities converge on the dysfunction in the locomotor regions primarily recruited for automatic motor regulation, particularly the MLR/PPN and the CLR, in PD patients with FoG. Dysfunction in these regions is paralleled by alterations in frontoparietal regions that support cognitive and executive functions as well as in parieto-occipital regions that support visuospatial processing. Furthermore, the communication between the locomotor circuits responsible for automatic gait control and the cognitive circuits responsible for higher-level volitional gait control is disrupted, as evidenced by diverse structural and functional cortico-subcortical disconnections in PD patients with FoG. A recent ALE *meta*-analysis of neuroimaging studies revealed that gait impairments in PD are associated with lower SMA and higher CLR activation (Gilat et al., 2019), corroborating the notion that disconnection between cortical and subcortical regions is a critical pathophysiological aspect of FoG.

The reviewed neuroimaging studies of FoG in PD do not converge on a single unifying pathophysiological model of FoG. On the one hand, structural MRI, and task-based and resting-state fMRI studies, reveal neuroanatomical/-physiological changes in PD patients with FoG that agree with the ‘perceptual dysfunction’ and ‘interference’ models of FoG. On the other hand, fNIRS studies, with their restricted focus on prefrontal regions, mainly support the ‘executive dysfunction’ model of FoG, and, to a lesser extent, the ‘perceptual dysfunction’ model of FoG. Importantly, some studies suggest that these models have a complementary role in explaining the pathophysiology of FoG across the different stages of FoG development in PD patients (Belluscio et al., 2019, Piramide et al., 2020). In particular, the different models of FoG can account for the diverse neural correlates of FoG that gradually emerge with the progression of FoG severity. The ‘interference’ and ‘executive dysfunction’ models can account for the often observed recruitment of frontoparietal regions in PD patients with mild FoG. In contrast, the ‘perceptual dysfunction’ model can account for the increased reliance on visuospatial resources to guide gait in PD patients with more severe FoG and concomitant deficits in executive functions. Note that this change from the reliance on frontostriatal networks in less severe FoG to reliance on visual associative networks in more severe FoG is following Braak’s staging of the pathological changes associated with PD, according to which damage progressively extends from the pontine tegmentum to the BG, the prefrontal and premotor areas, finally reaching the sensory association areas in the posterior brain (Braak et al., 2004). To further explore the differential recruitment of various compensatory strategies along with the progression of FoG symptoms, future studies are warranted that compare groups of PD patients with different FoG severity or that longitudinally compare PD patients with FoG at different phases of their disease progression.

Furthermore, the current pathophysiological models of FoG in PD do not consider putative different neural substrates underlying episodes of FoG that occur when initiating gait versus those FoG episodes that occur during walking. As discussed in the introduction, efficient execution of a motor plan necessitates coordinated sequential neural activity at the level of the BG, namely a timely inhibition, disinhibition, and finally, re-inhibition of a given motor plan (see Fig. 1). Therefore, a sustained inhibition via the hyper-direct pathway that persists abnormally long may hamper the initiation of gait and thus might contribute to FoG at gait initiation. In contrast, an abrupt re-inhibition via the indirect pathway before an ongoing movement is completed might account for FoG occurring during ongoing movement. Accordingly, the combination of reduced inhibition of the GPi/SNr complex via the direct pathway (pathological disinhibition) with either sustained baseline inhibition via the hyper-direct pathway or an early re-inhibition via the indirect pathway may account for FoG episodes that occur at the initiation of gait or during an ongoing movement, respectively.

The reported imaging studies of gait impairments in PD patients help identify target brain regions for potential treatment strategies. For instance, the pathophysiological importance of the SMA in developing FoG recently led researchers to explore the potential treatment effects of rTMS applied to this region. The authors reported normalization of abnormal connectivity patterns in the SMA following high-frequency rTMS, which alleviated FoG (Mi et al., 2020). The findings reported by Ehgoetz Martens et al. (2018) are probably the most relevant for developing novel treatment plans. These authors identified context-dependent triggers (motor, cognitive and limbic) that produce distinct and systematic impairments across different levels of the same locomotor network. The discovery of such unique neural signatures of FoG may shift PD treatment strategies from one-size-fits-all toward individualized treatment plans. For instance, PD patients with FoG triggered by cognitive deficits may benefit from a treatment plan that focuses on cognitive training to improve their cognitive faculties. In contrast, patients with affect-induced FoG may benefit from treatment that targets affective symptoms (like anxiety or depression). Furthermore, combining these individualized treatments with neuroimaging might allow monitoring treatment-induced changes in neural activity and connectivity directly. For example, VR paradigms can target the motor subtype of FoG in PD patients by implementing online feedback cues that assist and modulate sensorimotor functions.

The apparent disparity in the neurophysiological underpinnings of FoG in PD patients can be partially attributed to differences in adopted neuroimaging modalities, applied analysis methods, and implemented experimental paradigms. Additional limitations that further hinder the reproducibility and generalizability of findings concerning FoG in PD patients include the inclusion of PD patients with a wide spectrum of disease severity and duration, small sample sizes, and the lack of an extensive neuropsychological examination of PD patients with FoG. Remarkably, most studies supporting the ‘executive dysfunction’ and ‘perceptual dysfunction’ models of FoG fail to provide a thorough neuropsychological characterization of the cognitive and visuospatial deficits in the studied PD patients with FoG. Consequently, the PD patients’ cognitive and visuospatial deficits, which are relevant to the hypotheses derived from the models mentioned above, are not correlated with the neuroimaging findings. The variable medication state is another putative confound of imaging studies on FoG in PD. Still, too few imaging studies compared FoG in PD with both ON and OFF medication, which can shed further light on putative dopaminergic effects on FoG in PD (Wu and Hallett, 2005). Factorial study designs comparing PD patients with and without FoG in the ON versus OFF state are required to understanding the effect of levodopa treatment on FoG in PD and assessing potential interindividual differences in the responsiveness to levodopa treatment.

In conclusion, the present review highlights the significant imaging findings for freezing of gait (FoG) in PD patients and discusses the neural correlates in light of the different pathophysiological models of FoG. Although the imaging findings do not converge on a single pathophysiological model of FoG, evidence suggests that FoG in PD is associated with more than a single neural mechanism. The studies converge on highlighting functional decoupling between cortical and subcortical regions as well as abnormal activation patterns, particularly within frontoparietal and parieto-occipital regions and the striatum. The current review demonstrates that despite multiple pathophysiological insights across the different studies, systematic comparisons are limited due to differences in neuroimaging modalities and targeted brain mechanisms. Notably, the potential differential neural substrates underlying FoG during walking and FoG during gait initiation remain unaddressed. Thus, developing novel experimental paradigms that tackle this distinction is warranted. Besides, future studies should adopt multimodal neuroimaging and address the above-mentioned limitations that currently hamper comprehensive imaging studies of FoG in PD patients. These future imaging studies will hopefully permit the improvement of current treatment strategies and set the ground to devise novel treatments for FoG in PD that are tailored to the individual patient and thus more efficient.

## Declaration of Competing Interest

The authors declare that they have no known competing financial interests or personal relationships that could have appeared to influence the work reported in this paper.
