# Supplementary material for: Imaging the neural underpinnings of freezing of gait in Parkinson’s disease
Source: Neuroimage Clin. 2022 Jul 25;35:103123. doi: 10.1016/j.nicl.2022.103123 (PMC9421505; doi:10.1016/j.nicl.2022.103123)
Supplement: Supplementary Data 1 [file mmc1.docx]

**Table S1**: Ratings of the structural and resting-state functional connectivity studies investigating freezing of gait (FoG) in PD patients.

| **Author/Year** | **Sample** | **Clinical groups** | **FoG/gait assessment** | **Corrections of multiple comparisons** | **Control for differences in covariance** | **Experimental Design** | **Overall rating** |
| --- | --- | --- | --- | --- | --- | --- | --- |
| Bharti *et al.*  (2019a) | 15 FoG+  16 FoG-  16 HC  **(++)** | FoG+; HC; FoG-  **(++)** | FoG-Q **(+)**  TUG **(+)** | (FC): FWE cluster-level corrected at p<0.05, and for the group comparisons, Bonferroni corrected at p<0.025  (DTI): FWE corrected with TFCE at p<0.05 **(+)** | No significant (group) differences to control for. | Cross-sectional (HC > FoG+; HC > FoG-; FoG- > FoG+) **(+)**  Correlational (with FoG-Q scores) **(+)** | **9**  (=9-0) |
| Bharti *et al.* (2019b) | 15 FoG+  16 FoG-  16 HC **(+)** | FoG+; HC; FoG-  **(++)** | FoG-Q **(+)**  TUG **(+)** | FC (networks): FWE cluster-level corrected at p<0.05  **(+)** | Age, sex, and disease duration controlled for.  **(+)** | Cross-sectional (HC > PD, FoG+ > FoG-) **(+)**  Correlational (with FoG-Q scores)  **(+)** | **9**  (=9-0) |
| Canu *et al.* (2015);  *two samples* | 50 FoG+  25 FoG-  60 HC **(+)**  *(Combined numbers of two samples)* | FoG+; HC; FoG- **(++)** | FoG-Q **(+)**  TUG **(+)** | DTI & FC (RSN): FWE cluster-level corrected with TFCE using a significance threshold of p<0.05 **(+)** | Several clinical features not controlled for **(-)** | Cross-sectional (FoG- > FoG+; HC > FoG+; HC > FoG-) **(+)**  Correlational (with UPDRS-III) **(+)** | **7**  (=8-1) |
| Droby *et al.*  (2021) | 16 FoG+  15 FoG-  20 HC **(+)** | FoG+; HC; FoG-  **(++)** | FoG-Q **(+)** | Family-wise error corrected for cluster size (FWEc) with Monte Carlo simulation and p<0.001 **(+)** | Age, sex, and H&Y stage controlled for.  **(+)** | Cross-sectional (FoG- > FoG+; HC > FoG+; HC > FoG-) **(+)**  Correlational (with FoG-Q) **(+)** | **8**  (=8-0) |
| Fling *et al.* (2013) | 14 FoG+  12 FoG-  15 HC **(-)** | FoG+; HC; FoG-  **(++)** | FoG-Q **(+)**  TUG **(+)** | (ROI) Bonferroni corrected at p = 0.008 (0.05/6)  **(+)** | UPDRS-motor controlled for **(+)**, but not for MoCA, H&Y, and Stroop task **(-)** | Cross-sectional (HC > FoG-; HC > FoG+; FoG- > FoG+) **(+)**  Correlational (with Stroop task) **(+)** | **6**  (=8-2) |
| Fling *et al.* (2014) | 8 FoG+  7 FoG-  14 HC **(-)** | FoG+; HC; FoG-  **(++)** | New FoG-Q **(+)**  Gait task **(+)** | FC (between SMA and six sub-cortical ROIs) and DTI (SMA & STN/MLR): Tukey HSD corrected at p =< 0.05 **(+)** | UPDRS controlled for  **(+)** | Cross-sectional (FoG- > FoG+; HC > FoG+; HC > FoG-) **(+)**  Correlational (with new FoG-Q and behavioral measures of gait) **(++)** | **8**  (=9-1) |
| Jung *et al.*  (2020) | 26 FoG+  61 FoG-  27 HC **(+)** | FoG resistant, FoG vulnerable, HC **(++)** | Assessment of FoG by gait experts, precise criteria described **(+)** | Monte Carlo simulations corrected for p < 0.05/15 (uncorrected per voxel height threshold p<0.005 with a minimum cluster size of 134 voxels) **(+)** | Age, sex, education, UPDRS-III, and disease duration controlled for. **(+)** | Longitudinal design (5 years from MRI scan) **(+)**  Cross-sectional (FoG+/-, HC) **(+)**  Correlational (correlation time of FoG symptoms onset) **(+)** | **9**  (=9-0) |
| Lench *et al.* (2020) | 27 FoG+  27 FoG- **(+)** | FoG+, FoG-  **(+)** | New FoG-Q **(+)**  TUG **(+)** | (FC of PPN with whole brain) FWE-corrected at cluster level p< 0.05 (voxel threshold p < 0.01) **(+)** | LEDD and disease duration controlled for **(+)** | Cross-sectional **(+)**  Correlational (with TUG scores) **(+)** | **8**  (=8-0) |
| Lenka *et al.* (2015) | 15 FoG+  13 FoG-  30 HC **(++)** | FoG+, FoG-, HC  **(++)** | FoG-Q **(+)** | Whole-brain cluster-level FDR corrected at p<0.02  **(+)** | No significant differences to control for. | Cross-sectional (HC > FoG-; HC > FoG+; FoG- > FoG+) **(+)**  Correlational (with FoG-Q scores) **(+)** | **8**  (=8-0) |
| Miranda-Dominguez *et al.* (2020) | 13 FoG+  14 FoG-  16 HC **(- ; +)** | FoG+, FoG-, HC  **(++)** | FoG-Q **(+)**  Gait task **(+)** | (ROI) FDR corrected at p<0.05  (whole brain) corrected at alpha –log p=1.3 and z > 1.96 using TFCE **(+)** | No significant differences to control for. | Cross-sectional (HC > FoG-; HC > FoG+; FoG- > FoG+) **(+)**  Correlation (behavioral measures of gait outside scanner) **(+)** | **7**  (=8-1) |
| Pietracupa *et al.* (2018) | 21 FoG+  16 FoG-  19 HC **(+)** | FoG+, FoG-, HC  **(++)** | FoG-Q **(+)**  TUG **(+)** | DTI: Bonferroni corrected at  p =< 0.012 (0.05/4)  **(++)** | Age, sex, and disease duration controlled for **(+)** | Cross-sectional (HC > FoG+; HC > FoG-) **(-)**  Correlational (with FoG-Q and neuropsychological tests) **(++)** | **9**  (=10-1) |
| Potvin-Desrochers *et al.* (2019) | 14 FoG+  13 FoG-  **(-)** | FoG+, FoG-  **(+)** | New FoG-Q **(+)** | Gaussian random field theory with cluster-thresholding of z > 2.3 and a cluster significance of p<0.05 corrected **(+)** | Differences in H&Y stage and HADS depression scores not controlled for **(-)** | Cross-sectional (FoG+ > FoG-)  **(+)** | **2**  (=4-2) |
| Steidel *et al.* (2021) | 27 FoG+  32 FoG-  **(+)** | FoG+, FoG-  **(+)** | FoG-Q **(+)** | Cluster-level FWE-corrected at p < 0.05, and correlations were FDR corrected at p< 0.05 **(+)** | Significant differences in BDI-II, PDQ-39, and visuospatial scores not controlled for **(-)** | Cross-sectional (FoG+ > FoG-, FoG+ < FoG-) **(+)**  Correlational (dopaminergic degeneration & FoG-Q) **(++)** | **6**  (=7-1) |
| Tessitore *et al.* (2012) | 16 FoG+  13 FoG-  15 HC  **(-)** | FoG+, FoG-, HC  **(++)** | FoG-Q **(+)** | Cluster level correction at p<0.05 (initial voxel-level threshold at p = 0.01 and minimum cluster size estimated by 1000 Monte Carlo simulations) **(+)** | Significant differences on cognitive tests not controlled for **(-)** | Cross-sectional (HC > all PD; FoG- > FoG+) **(+)**  Correlational (with FoG-Q scores) **(+)** | **4**  (=6-2) |
| Vervoort *et al.* (2016) | 13 FoG+  60 FoG-  20 HC  **(+)** | FoG+, FoG-, HC  **(++)** | New FoG-Q **(+)**  Gait task **(+)** | (ROI) FDR corrected at p = 0.05 **(+)** or uncorrected at p = 0.001 **(-)** | Age, disease duration, and UPDRS controlled for **(+)** | Cross-sectional (HC > all PD; FoG- > FoG+) **(+)**  Correlational (with behavioral measures of turning) **(+)** | **8**  (=9-1) |
| Wang *et al.* (2016) | 14 FoG+  16 FoG-  16 HC  **(+)** | FoG+, FoG-, HC  **(++)** | FoG-Q **(+)**  TUG **(+)** | DTI: statistical maps thresholded at p < 0.005, and FWE corrected at cluster level using TFCE  FC (ROI): Voxel-level p<0.01, cluster size > 9 voxels, AlphaSim corrected at p < 0.05 **(+)** | Age, sex, and education controlled for **(+)** | Cross-sectional (HC > FoG-; HC > FoG+; FoG- > FoG+) **(+)** | **8**  (=8-0) |
| Wang *et al.* (2021) | 25 FoG+  25 FoG-  25 HC  **(++)** | FoG+, FoG-, HC  **(++)** | New FoG-Q **(+)**  Turning task **(+)** | FWE-corrected with a cluster defining threshold of p<0.001 and a corrected cluster significance of p<0.05 **(+)** | No significant differences to control for. | Cross-sectional (HC > FoG-; HC > FoG+; FoG- > FoG+) **(+)**  Correlational (with new FoG-Q) **(+)** | **9**  (=9-0) |
| Yu *et al.* (2021) | 20 FoG+  21 FoG-  18 HC **(+)** | FoG+, FoG-, HC  **(++)** | FoG-Q **(+)**  TUG **(+)** | (FC between DAN and seven networks) FDR corrected at p<0.05 (also DTI)  **(+)** | Significant differences in MoCA, FAB, TMT, and CDT were not controlled for.  **(-)** | Cross-sectional design (HC > FoG-; HC > FoG+; FoG- > FoG+) **(+)**  Correlational (with FAB, CDT and TUG) **(++)** | **8**  (=9-1) |

**Table S1**: **Ratings of the structural and resting-state functional connectivity studies investigating freezing of gait (FoG) in PD patients.**

To assess the quality of evidence provided by the different imaging studies, we systematically graded each reported study based on six pre-defined criteria: studied sample, clinical groups, FoG/gait assessment, correction for multiple comparisons, control for differences in covariates, and experimental design.

**Criteria for studied sample**: A study gains one point if the sample is sufficiently large for an imaging study and an additional point if the studied groups are matched for demographic and neuropsychological parameters. Accordingly, a point is deducted if the sample size is not large enough. For the purpose of the current review, a sample is considered sufficiently large for an imaging study if it includes a minimum of 15 subjects per group (or correspondingly, at least 30/ 45 subjects when two/ three groups were investigated). Thus, the maximum number of points a study can gain for this criterion is 2 points.

**Criteria for clinical groups:** A study gains one point if the sample includes PD patients with FoG and a single control group of either healthy controls or PD patients without FoG. An additional point is granted if the study includes PD patients with FoG and two control groups, i.e., PD patients without FoG and healthy controls. However, a point is deducted if the sample does not include PD patients with FoG. Thus, the maximum number of points a study can gain for this criterion is 2 points.

**Criteria for FoG/gait assessment:** A study gains one point if FoG is assessed using a subjective measure (e.g., FoG-Questionnaire) and an additional point if FoG (or other gait impairments) are assessed using an objective measure. However, a point is deducted if the study does not include any assessment of FoG. Thus, the maximum number of points a study can gain for this criterion is 2 points.

**Criteria for correction of multiple comparisons:** A study gains a point if the reported imaging findings are corrected for multiple comparisons at the cluster level or ROI level. However, a point is deducted if the study reports uncorrected imaging results. Thus, the maximum number of points a study can gain for this criterion is 1 point.

**Criteria for control for differences in covariance:** A study gains a point if the imaging results are controlled for neuropsychological or demographical measures that are not matched between the examined groups. However, a point is deducted if the study does not control for detected differences in measured variables (or for any clinical variable if the study contains only one clinical group). Note that no points are gained or deducted for studies with matching samples as a point would already be given for the respective study under the ‘sample’ criterion. Thus, the maximum number of points a study can gain for this criterion is 1 point.

**Criteria for experimental design:** (i) If the design is cross-sectional, the study gains one point. (ii) If the design is correlational, the study gains one point for a correlation with subjective measures of FoG (e.g., FoG-Q), and one additional point for a correlation with objective measures of FoG/gait (e.g., Timed up and go task), and one additional point for a correlation with cognitive measures. (iii) If the design is comparative, the study gains one point for a comparison across task conditions (e.g., in task-based studies) and an additional point for a comparison across medical conditions (e.g., medication state). Thus, the maximum number of points a study can gain for this criterion is 5 points. Note that one study (Jung et al.; 2020) gained an additional point for the experimental design criterion for employing a longitudinal design.

Studies are listed in alphabetical order. BDI-II: Beck depression inventory, CDT: clock drawing test, DAN: dorsal attention network, DTI: diffusion tensor imaging, FAB: frontal assessment battery, FC: Functional connectivity, FDR: false discovery rate, FoG+: PD patients with freezing of gait, FoG-: PD patients without freezing of gait, FoG-Q: freezing of gait questionnaire, FWE: family-wise error, HADS: Hamilton anxiety and depression scale, HC: healthy control participants, H&Y: Hoehn & Yahr, LEDD: levodopa equivalent daily dose, MLR: mesencephalic locomotor region, MoCA: Montreal cognitive assessment, PD: Parkinson’s disease, PDQ-39: Parkinson’s disease questionnaire [item assessing the quality of life], PPN: pedunculopontine nucleus, ROI: region of interest, RSN: resting-state network, SMA: supplementary motor area, STN: subthalamic nucleus, TFCE: threshold-free cluster enhancement, TMT: trail making test, TUG: timed up and go, UPDRS: Unified Parkinson’s disease rating scale.

**Table S2**: Ratings of the task-based fMRI studies assessing freezing of gait (FoG) in Parkinson’s disease (PD) patients.

| **Author/Year** | **Sample** | **Clinical groups** | **FoG/gait assessment** | **Corrections of multiple comparisons** | **Control for differences in covariance** | **Experimental Design** | **Overall rating** |
| --- | --- | --- | --- | --- | --- | --- | --- |
| Cremers *et al.* (2012) | 15 PD  15 HC  **(++)** | PD; HC  **(-)** | 25-meters gait **(+)**  No FoG assessment **(-)** | Peak-cluster level and FWE corrected at p<0.05  **(+)** | No variables to control for | Cross-sectional (HC > PD) **(+)**  Comparative (gait > standing) **(+)**  Correlational (direct with MI and indirect with 25-meters) **(++)** | **6**  (=8-2) |
| Ehgoetz-Martens *et al.* (2018) | 20 FoG+  **(+)** | FoG+  **(+)** | FoG-Q **(+)**  Min 5 freezing events during the VR task **(+)** | (ROI) Non-linear permutation testing (5000) with results thresholded using a large effect size (d > 0.8) **(+)** | Clinical variables not controlled for in GLM **(-)** | Comparative (freezing > normal walking; low vs. high load) **(+)**  Correlational (direct with motor arrests; indirect with cognitive tests) **(++)** | **7**  (=8-1) |
| Gilat *et al.* (2015) | 17 FoG+  10 FoG-  **(-)** | FoG+; FoG-  **(+)** | FoG-Q **(+)**  TUG **(+)** | (ROI) FDR corrected at p<0.05 with a threshold value 3 **(+)** | MoCA and HADS depression & anxiety scores controlled for **(+)** | Cross-sectional (FoG- > FoG+) **(+)**  Comparative (walking > turning) **(+)**  Correlational (direct with motor arrests) **(+)** | **7**  (=8-1) |
| Huang *et al.,* (2021) | 22 FoG+  15 FoG-  15 HC  **(+)** | FoG+; FoG-; HC  **(++)** | New FoG-Q **(+)**  5 meters gait **(+)** | False discovery rate (FDR) at voxel level corrected at p<0.05 and ROI analysis at p < 0.05 **(+)**  Some results were reported at uncorrected p < 0.001 **(-)** | H&Y staging controlled for **(+)** | Cross-sectional (FoG+ > HC; FoG+ > FoG; FoG- > HC) **(+)**  Comparative (normal gait > FoG; gait > turning) **(+)**  Correlational (direct MI) **(+)** | **9**  (=10-1) |
| Matar *et al.*  (2019) | 19 FoG+  **(+)** | FoG+  **(+)** | FoG-Q **(+)** | Uncorrected alpha level of 0.005 and a cluster threshold of 10 **(-)** | Clinical variables not controlled for **(-)** | Comparative (ON vs. OFF; wide vs. narrow doors) **(++)**  Correlational (direct with motor arrests) **(+)** | **4**  (=6-2) |
| Peterson *et al.* (2014a) | 9 FoG+  9 FoG-  **(-)** | FoG+; FoG-  **(+)** | New FoG-Q **(+)**  gait task **(+)** | ROI spearman rho correlation uncorrected at p < 0.05 **(-)** | UPDRS-III and average gait imagery speed for each participant controlled for **(+)** | Cross-sectional (FoG+ > FoG-) **(+)**  Comparative (stand > gait) **(+)**  Correlational (direct with MI, indirect with new FoG-Q and gait task) **(+++)** | **7**  (=9-2) |
| Peterson *et al.* (2014b) | 19 PD (9 FoG+)  20 HC  **(+)** | PD; HC  **(-)** | New FoG-Q **(+)**  gait task **(+)** | ROI spearman rho correlation uncorrected at p < 0.05 **(-)** | Average MI times controlled for **(+)** | Cross-sectional (PD > HC) **(+)**  Comparative (stand > gait) **(+)**  Correlational (direct with MI, indirect with gait task) **(++)** | **6**  (=8-2) |
| Piramede *et al.* (2020) | 17 FoG+,  10 FoG-  18 HC  **(+)** | FoG+ (mild, moderate); FoG-; HC  **(++)** | FoG-Q **(+)**  TUG, 10 meters walking task **(+)** | Corrected at cluster level using small volume correction (10 mm radius) with a cutoff value of p < 0.05 **(+)** | MCI not controlled for  **(-)** | Cross-sectional (FoG+ > FoG-; FoG-mild > FoG-moderate; FoG+ > HC) **(+)**  Correlational (direct with gait measures of dorsal and plantar foot flexion, indirect with clinical measures) **(++)** | **8**  (=9-1) |
| Shine *et al.*  (2013a) | 18 FoG+  **(+)** | FoG+  **(+)** | FoG-Q **(+)**  TUG **(+)** | (mixed block event analysis) Uncorrected for multiple comparisons at p < 0.001 with a cluster threshold of 10 voxels **(-)**  ROI FWE-corrected at p<0.05 **(+)** | Clinical variables not controlled for **(-)** | Comparative (high vs low, walking vs. freezing) **(+)**  Correlational (indirect with FoG-Q, direct with motor arrests) **(++)** | **6**  (=8-2) |
| Shine *et al.*  (2013b) | 10 FoG+  10 FoG-  **(-)** | FoG+; FoG-  **(+)** | FoG-Q **(+)**  TUG **(+)** | Bonferroni corrected at p = 0.01 (network BOLD) and at p = 0.005 (network connectivity) **(+)** | Clinical variables not controlled for **(-)** | Cross-sectional (FoG- > FoG+) **(+)**  Comparative (high vs. low; ON vs. OFF; walking, pre/post arrest) **(++)**  Correlational (direct correlation with motor arrests) **(+)** | **6**  (=8-2) |
| Shine *et al.*  (2013c) | 14 FoG+  15 FoG-  **(+)** | FoG+; FoG-  **(+)** | FoG-Q **(+)**  TUG **(+)** | ROI Corrected with a false detection rate of p < 0.05  **(+)** | HADS scores controlled for **(+)** | Cross-sectional (FoG- > FoG+) **(+)**  Comparative (high vs. low; walking vs. freezing) **(+)**  Correlational (direct correlation with motor arrests) **(+)** | **9**  (=9-0) |
| Snjiders *et al.* (2011) | 12 FoG+  12 FoG-  21 HC  **(+)** | FoG+; FoG-; HC  **(++)** | New FOG-Q **(+)**  gait task **(+)** | FWE corrected at p< 0.05 and intensity threshold t > 3.4 at the cluster level **(+)** | Clinical variables not controlled for **(-)** | Cross-sectional (PD > HC; FoG+ > FoG-) **(+)**  Comparative (path width and length) **(+)**  correlational (direct with MI duration, indirect with new FoG-Q) **(++)** | **9**  (=10-1) |
| Wai *et al.* (2012) | 13 PD  13 HC-old  14 HC- young  **(-)** | PD; HC old; HC young  **(-)** | No FoG assessment **(-)** | Family wise error corrected at p< 0.05 in cluster level **(+)** | H&Y and UPDRS controlled for **(+)** | Cross-sectional (PD > HC) **(+)**  Comparative (gait initiation/termination, OA) **(+)**  correlational (direct with MI duration) **(+)** | **2**  (=5-3) |

**Table S2**: **Ratings of the task-based fMRI studies assessing freezing of gait (FoG) in Parkinson’s disease (PD) patients.**

To assess the quality of evidence provided by the different imaging studies, we systematically graded each reported study based on six pre-defined criteria: studied sample, clinical groups, FoG/gait assessment, correction for multiple comparisons, control for differences in covariates, and experimental design.

**Criteria for studied sample**: A study gains one point if the sample is sufficiently large for an imaging study and an additional point if the studied groups are matched for demographic and neuropsychological parameters. Accordingly, a point is deducted if the sample size is not large enough. For the purpose of the current review, a sample is considered sufficiently large for an imaging study if it includes a minimum of 15 subjects per group (or correspondingly, at least 30/ 45 subjects when two/ three groups were investigated). Thus, the maximum number of points a study can gain for this criterion is 2 points.

**Criteria for clinical groups:** A study gains one point if the sample includes PD patients with FoG and a single control group of either healthy controls or PD patients without FoG. An additional point is granted if the study includes PD patients with FoG and two control groups, i.e., PD patients without FoG and healthy controls. However, a point is deducted if the sample does not include PD patients with FoG. Thus, the maximum number of points a study can gain for this criterion is 2 points.

**Criteria for FoG/gait assessment:** A study gains one point if FoG is assessed using a subjective measure (e.g., FoG-Questionnaire) and an additional point if FoG (or other gait impairments) are assessed using an objective measure. However, a point is deducted if the study does not include any assessment of FoG. Thus, the maximum number of points a study can gain for this criterion is 2 points.

**Criteria for correction of multiple comparisons:** A study gains a point if the reported imaging findings are corrected for multiple comparisons at the cluster level or ROI level. However, a point is deducted if the study reports uncorrected imaging results. Thus, the maximum number of points a study can gain for this criterion is 1 point.

**Criteria for control for differences in covariance:** A study gains a point if the imaging results are controlled for neuropsychological or demographical measures that are not matched between the examined groups. However, a point is deducted if the study does not control for detected differences in measured variables (or for any clinical variable if the study contains only one clinical group). Note that no points are gained or deducted for studies with matching samples as a point would already be given for the respective study under the ‘sample’ criterion. Thus, the maximum number of points a study can gain for this criterion is 1 point.

**Criteria for experimental design:** (i) If the design is cross-sectional, the study gains one point. (ii) If the design is correlational, the study gains one point for a correlation with subjective measures of FoG (e.g., FoG-Q), and one additional point for a correlation with objective measures of FoG/gait (e.g., Timed up and go task), and one additional point for a correlation with cognitive measures. (iii) If the design is comparative, the study gains one point for a comparison across task conditions (e.g., in task-based studies) and an additional point for a comparison across medical conditions (e.g., medication state). Thus, the maximum number of points a study can gain for this criterion is 5 points. Note that one study (Jung et al.; 2020) gained an additional point for the experimental design criterion for employing a longitudinal design.

Studies are listed in alphabetical order. BDI-II: Beck depression inventory, CDT: clock drawing test, DAN: dorsal attention network, DTI: diffusion tensor imaging, FAB: frontal assessment battery, FC: Functional connectivity, FDR: false discovery rate, FoG+: PD patients with freezing of gait, FoG-: PD patients without freezing of gait, FoG-Q: freezing of gait questionnaire, FWE: family-wise error, HADS: Hamilton anxiety and depression scale, HC: healthy control participants, H&Y: Hoehn & Yahr, LEDD: levodopa equivalent daily dose, MLR: mesencephalic locomotor region, MoCA: Montreal cognitive assessment, PD: Parkinson’s disease, PDQ-39: Parkinson’s disease questionnaire [item assessing the quality of life], PPN: pedunculopontine nucleus, ROI: region of interest, RSN: resting-state network, SMA: supplementary motor area, STN: subthalamic nucleus, TFCE: threshold-free cluster enhancement, TMT: trail making test, TUG: timed up and go, UPDRS: Unified Parkinson’s disease rating scale.

Studies are listed in alphabetical order. BOLD: blood oxygen level dependent, FC: Functional connectivity, FDR: false discovery rate, FoG+: PD patients with freezing of gait, FoG-: PD patients without freezing of gait, FoG-Q: freezing of gait questionnaire, FWE: family-wise error, GLM: general linear model, HADS: Hamilton anxiety and depression scale, HC: healthy control participants, H&Y: Hoehn & Yahr, MCI: mild cognitive impairment, MI: motor imagery, MoCA: Montreal cognitive assessment, OA: obstacle avoidance, PD: Parkinson’s disease, ROI: region of interest, TUG: timed up and go, UPDRS: Unified Parkinson’s disease rating scale, VR: virtual reality.

**Table S3**: Ratings of the activation studies of actual gait in PD patients employing functional near-infrared spectroscopy (fNIRS)

| **Author/Year** | **Sample** | **Clinical groups** | **FoG/gait assessment** | **Corrections of multiple comparisons** | **Control for differences in covariance** | **Experimental Design** | **Overall rating** |
| --- | --- | --- | --- | --- | --- | --- | --- |
| Belluscio *et al.* (2019) | 15 FoG+  17 FoG-  8 HC  **(+)** | FoG+, FoG-, HC  **(++)** | New FoG-Q **(+)** | Bonferroni corrected at p < 0.01 **(+)** | Differences in TMT and MoCA scores not controlled for **(-)** | Cross-sectional **(+)**  Comparative (single vs. dual task) **(+)**  Correlational (indirect with visuospatial scores, new FoG-Q, and direct with gait measures) **(+++)** | **9**  (=10-1) |
| Dagan *et al.*  (2020) | 40 FoG+  **(+)** | FoG+  **(+)** | New FoG-Q **(+)** | Benjamini Hochberg corrected at p < 0.05 **(+)** | Age, sex, UPDRS controlled for **(+)** | Comparative (single vs. dual task, and ON vs. OFF medication) **(++)**  Correlational (direct with gait measures) **(+)** | **8**  (=8-0) |
| Maidan *et al.* (2016) | 68 PD  38 older HC  **(+)** | PD, HC  **(-)** | No FoG assessment **(-)**  2 minutes walking test **(+)** | Bonferroni corrected at p =< 0.05 **(+)** | Age, sex, executive functions controlled for **(+)** | Cross-sectional **(+)**  Comparative (single, dual, OA) **(+)**  Correlational (direct with gait measures) **(+)** | **5**  (=7-2) |
| Nieuwhof *et al.* (2016) | 12 PD  **(-)** | PD  **(-)** | No FoG assessment  **(-)** | Uncorrected p < 0.05 **(-)** | Does not control for any variables **(-)** | Comparative (single vs. dual) **(+)**  Correlational (direct with gait measures) **(+)** | **-3**  (=2-5) |
| Orcioli-Silva *et al.* (2020) | 17 PD-PIGD  19 PD-TD  **(++)** | PD-PIGD, PD-TD  **(-)** | No FoG assessment  **(-)** | Tukey post-hoc test at p<0.05 **(+)** | No differences to control for | Cross-sectional (TD vs. PIGD) **(+)**  Comparative (walking vs. OA) **(+)**  Correlational (direct with gait measures) **(+)** | **4**  (=6-2) |
| Ranchet *et al.*  (2020) | 18 PD  18 HC  **(+)** | PD, HC  **(-)** | No FoG assessment  **(-)** | Bonferroni corrected at p<0.05 **(+)** | Separate linear models to predict hemoglobin levels using all demographic and clinical variables **(+)** | Cross-sectional (PD vs. HC) **(+)**  Comparative (single vs. dual walking) **(+)**  Correlational (direct with gait measures, indirect with TMT, DSST, and Stroop test) **(++)** | **5**  (=7-2) |
| Stuart *et al.* (2020) | 19 FoG-  **(+)** | FoG-  **(+)** | FOG-Q (FoG+ were excluded) **(+)** | Uncorrected p < 0.05 **(-)** | Cognitive measures not controlled for **(-)** | Comparative (single vs. dual walking, and OFF vs. ON medication) **(++)**  Correlational (direct with gait measures) **(+)** | **4**  (=6-2) |
| Thumm *et al.* (2018) | 20 PD  **(+)** | PD  **(-)** | No FoG assessment  **(-)** | Uncorrected p < 0.05 **(-)** | Separate linear model to predict hemoglobin levels using clinical variables **(+)** | Comparative (over-ground vs. treadmill) **(+)**  Correlational (direct with gait measures) **(+)** | **1**  (=4-1) |
| Vitorio *et al.* (2020) | 24 FoG+  23 FoG-  **(+)** | FoG+; FoG-  **(+)** | New FoG-Q **(+)** | Uncorrected p < 0.05 **(-)** | Clinical and cognitive variables controlled for **(+)** | Cross-sectional (FoG+ vs. FoG-) **(+)**  Comparative (single vs. dual walking) **(+)**  Correlational (indirect with new FoG-Q, direct with gait measures) **(++)** | **7**  (=8-1) |

**Table S3**: **Ratings of the activation studies of actual gait in PD patients employing functional near-infrared spectroscopy (fNIRS)**

To assess the quality of evidence provided by the different imaging studies, we systematically graded each reported study based on six pre-defined criteria: studied sample, clinical groups, FoG/gait assessment, correction for multiple comparisons, control for differences in covariates, and experimental design.

**Criteria for studied sample**: A study gains one point if the sample is sufficiently large for an imaging study and an additional point if the studied groups are matched for demographic and neuropsychological parameters. Accordingly, a point is deducted if the sample size is not large enough. For the purpose of the current review, a sample is considered sufficiently large for an imaging study if it includes a minimum of 15 subjects per group (or correspondingly, at least 30/ 45 subjects when two/ three groups were investigated). Thus, the maximum number of points a study can gain for this criterion is 2 points.

**Criteria for clinical groups:** A study gains one point if the sample includes PD patients with FoG and a single control group of either healthy controls or PD patients without FoG. An additional point is granted if the study includes PD patients with FoG and two control groups, i.e., PD patients without FoG and healthy controls. However, a point is deducted if the sample does not include PD patients with FoG. Thus, the maximum number of points a study can gain for this criterion is 2 points.

**Criteria for FoG/gait assessment:** A study gains one point if FoG is assessed using a subjective measure (e.g., FoG-Questionnaire) and an additional point if FoG (or other gait impairments) are assessed using an objective measure. However, a point is deducted if the study does not include any assessment of FoG. Thus, the maximum number of points a study can gain for this criterion is 2 points.

**Criteria for correction of multiple comparisons:** A study gains a point if the reported imaging findings are corrected for multiple comparisons at the cluster level or ROI level. However, a point is deducted if the study reports uncorrected imaging results. Thus, the maximum number of points a study can gain for this criterion is 1 point.

**Criteria for control for differences in covariance:** A study gains a point if the imaging results are controlled for neuropsychological or demographical measures that are not matched between the examined groups. However, a point is deducted if the study does not control for detected differences in measured variables (or for any clinical variable if the study contains only one clinical group). Note that no points are gained or deducted for studies with matching samples as a point would already be given for the respective study under the ‘sample’ criterion. Thus, the maximum number of points a study can gain for this criterion is 1 point.

**Criteria for experimental design:** (i) If the design is cross-sectional, the study gains one point. (ii) If the design is correlational, the study gains one point for a correlation with subjective measures of FoG (e.g., FoG-Q), and one additional point for a correlation with objective measures of FoG/gait (e.g., Timed up and go task), and one additional point for a correlation with cognitive measures. (iii) If the design is comparative, the study gains one point for a comparison across task conditions (e.g., in task-based studies) and an additional point for a comparison across medical conditions (e.g., medication state). Thus, the maximum number of points a study can gain for this criterion is 5 points. Note that one study (Jung et al.; 2020) gained an additional point for the experimental design criterion for employing a longitudinal design.

Studies are listed in alphabetical order. BDI-II: Beck depression inventory, CDT: clock drawing test, DAN: dorsal attention network, DTI: diffusion tensor imaging, FAB: frontal assessment battery, FC: Functional connectivity, FDR: false discovery rate, FoG+: PD patients with freezing of gait, FoG-: PD patients without freezing of gait, FoG-Q: freezing of gait questionnaire, FWE: family-wise error, HADS: Hamilton anxiety and depression scale, HC: healthy control participants, H&Y: Hoehn & Yahr, LEDD: levodopa equivalent daily dose, MLR: mesencephalic locomotor region, MoCA: Montreal cognitive assessment, PD: Parkinson’s disease, PDQ-39: Parkinson’s disease questionnaire [item assessing the quality of life], PPN: pedunculopontine nucleus, ROI: region of interest, RSN: resting-state network, SMA: supplementary motor area, STN: subthalamic nucleus, TFCE: threshold-free cluster enhancement, TMT: trail making test, TUG: timed up and go, UPDRS: Unified Parkinson’s disease rating scale.

Studies are listed in alphabetical order. DSST: digit symbol substitution test, FoG+: PD patients with freezing of gait, FoG-: PD patients without freezing of gait, FoG-Q: freezing of gait questionnaire, HC: healthy Control participants, H&Y: Hoehn & Yahr, MoCA: Montreal cognitive assessment, OA: obstacle avoidance, PD: Parkinson’s disease, PIGD: postural instability gait disorder, TD: tremor dominant, TMT: trail making test, UPDRS: Unified Parkinson’s disease rating scale.
